# A novel monoclonal antibody against 6-sulfo sialyl Lewis x glycans attenuates murine allergic rhinitis by suppressing Th2 immune responses

**DOI:** 10.1038/s41598-023-43017-w

**Published:** 2023-09-21

**Authors:** Wei Liu, Wei Xiong, Wenxin Liu, Jotaro Hirakawa, Hiroto Kawashima

**Affiliations:** Laboratory of Microbiology and Immunology, Graduate School of Pharmaceutical Sciences, Chiba University, 1-8-1 Inohana, Chuo-Ku, Chiba, 260-8675 Japan

**Keywords:** Glycobiology, Lymph node

## Abstract

Lymphocyte homing is mediated by the interaction between L-selectin on lymphocytes and its glycoprotein ligands modified with 6-sulfo sialyl Lewis x (6-sulfo sLe^x^) glycans on high endothelial venules (HEVs) in peripheral lymph nodes (PLNs). However, the lack of specific antibodies reactive with both human and mouse 6-sulfo sLe^x^ has limited our understanding of its function in vivo. Here, we generated a novel monoclonal antibody, termed SF1, that specifically reacts with 6-sulfo sLe^x^ expressed on HEVs in both species in a manner dependent on sulfate, fucose, and sialic acid modifications. Glycan array and biolayer interferometry analyses indicated that SF1 specifically bound to 6-sulfo sLe^x^ with a dissociation constant of 6.09 × 10^–9^ M. SF1 specifically bound to four glycoproteins from PLNs corresponding to the molecular sizes of L-selectin ligand glycoproteins. Consistently, SF1 inhibited L-selectin-dependent lymphocyte rolling on 6-sulfo sLe^x^-expressing cells ex vivo and lymphocyte homing to PLNs and nasal-associated lymphoid tissues in vivo. Furthermore, SF1 significantly attenuated ovalbumin-induced allergic rhinitis in mice in association with significant suppression of Th2 immune responses. Collectively, these results suggest that SF1 can be useful for the functional analysis of 6-sulfo sLe^x^ and may potentially serve as a novel therapeutic agent against immune-related diseases.

## Introduction

**Figure 1:**
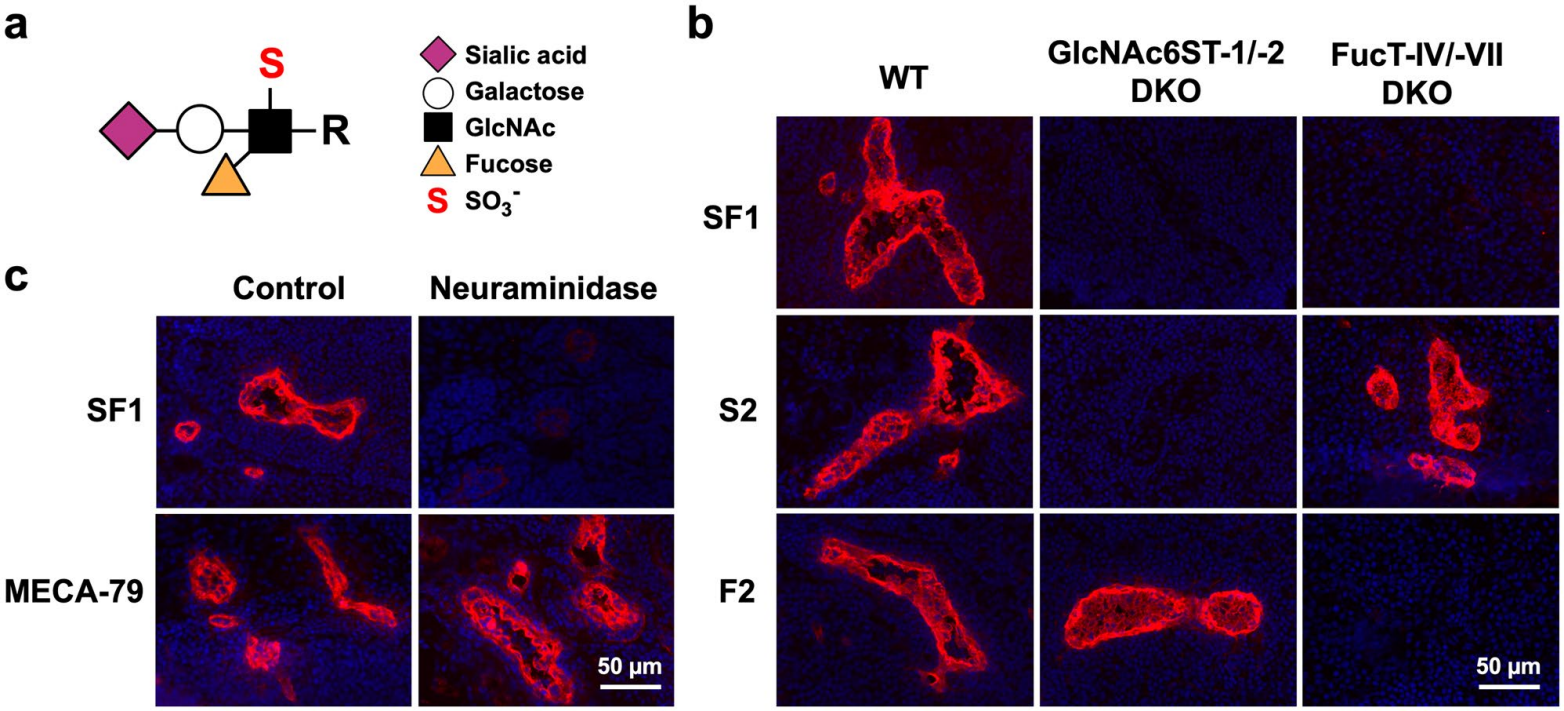
Immunofluorescence of PLN tissue sections from various strains of mice with anti-glycan mAbs. (**a**) Structure of 6-sulfo sLe^x^. (**b**) Immunofluorescence. Frozen sections of PLNs from C57BL/6 WT, GlcNAc6ST-1/-2 DKO, and FucT-IV/-VII DKO mice were incubated with biotinylated SF1, S2, or F2, followed by incubation with streptavidin-Alexa Fluor 594 (*red*) and DAPI (*blue*). (**c**) Effects of neuraminidase on mAb binding. Frozen sections of PLNs from C57BL/6 WT mice that had been treated with (*Neuraminidase*) or without (*Control*) α2-3,6,8 neuraminidase were stained with biotinylated SF1 or MECA-79 as in (**b**). *Bars*, 50 µm.

Lymphocytes continuously circulate in the body via the blood and lymph to monitor for foreign antigens^1–3^. Lymphocyte homing is a phenomenon in which circulating lymphocytes in peripheral blood migrate to secondary lymphoid organs, such as peripheral lymph nodes (PLNs), where immune responses occur. Lymphocyte homing to PLNs is critically dependent on the interaction between the homing receptor, L-selectin, expressed on the surface of lymphocytes and 6-sulfo sialyl Lewis x (6-sulfo sLe^x^; sialic acidα2-3Galβ1-4[Fucα1-3(sulfo-6)]GlcNAcβ1-R; Fig. 1a) expressed on the surface of specialized endothelial venules, called high endothelial venules (HEVs), as revealed by studies using sialidase^4,5^ and in α2,3-sialyltransferase-deficient^6^, α1,3-fucosyltransferase (FucT)-IV and -VII double-deficient (FucT-IV/-VII DKO)^7^, and *N*-acetylglucosamine-6-*O*-sulfotransferase (GlcNAc6ST)-1 and -2 double-deficient (GlcNAc6ST-1/-2 DKO) mice^8,9^. The structural difference between 6-sulfo sLe^x^ expressed in humans and mice is as follows: 6-sulfo sLe^x^ expressed in humans is exclusively modified with *N*-acetylneuraminic acid (Neu5Ac) because of the lack of the gene encoding CMP-Neu5Ac hydroxylase (Cmah) in humans^10^ which converts CMP-Neu5Ac to CMP-Neu5Gc, whereas that expressed in mice is modified with *N*-glycolylneuraminic acid (Neu5Gc) due to the expression of Cmah^11^. To date, the lack of specific antibodies reactive with both human and mouse 6-sulfo sLe^x^ has limited our understanding of its expression and functions in lymphocyte homing and immune-related diseases.

MECA-79^12^, a mAb widely used as a marker for human and mouse HEVs in PLNs, binds to 6-sulfo sLe^x^ only when it is present on a specific glycan core structure and recognizes extended core 1 *O*-glycans containing 6-sulfo *N*-acetyllactosamine (6-sulfo sialyl LacNAc, sialic acidα2-3Galβ1-4GlcNAcβ1-R) as its minimum epitope^13^. Previously developed mAbs, S1 and S2, also bind to 6-sulfo sLe^x^, but they recognize *O*-glycans containing 6-sulfo LacNAc and *N*- and *O*-glycans containing 6-sulfo LacNAc, respectively, as their minimum epitopes^14^. Similarly, mAb CL40 is reactive with 6-sulfo sLe^x^, which binds to both *N*- and *O*-glycans containing 6-sulfo LacNAc as its epitope^15^. MAbs F1 and F2 also bind to 6-sulfo sLe^x^, but recognize *N*- and *O*-glycans containing sialyl Lewis x (sLe^x^; sialic acidα2-3Galβ1-4[Fucα1-3]GlcNAcβ1-R) as their minimum epitopes^16^. Although these mAbs can react with both human and mouse 6-sulfo sLe^x^ expressed on HEVs and with a portion of the 6-sulfo sLe^x^ structure as their epitopes, none of them are specific to 6-sulfo sLe^x^. One mAb, G152, reacts specifically with the 6-sulfo sLe^x^ structure expressed in humans^17^; however, this mAb cannot bind to 6-sulfo sLe^x^ expressed in mice, and thus its effects on lymphocyte homing and immune-related diseases in mice cannot be determined. This is possibly because G152 selectively binds to glycans modified with Neu5Ac expressed in humans, whereas it fails to bind to glycans modified with Neu5Gc that is abundantly expressed in mice.

We previously established an efficient method for the generation of anti-glycan mAbs using glycosyltransferase- or sulfotransferase-deficient mice, based on the high immunogenicity of the missing glycans in mutant mice^14,16,18^. In addition, by immunizing mutant mice with transfectants overexpressing not only the missing enzymes but also Cmah^11^, we were able to generate mAbs reactive with both humans and mice. Based on this methodology, we herein generated a novel anti-6-sulfo sLe^x^ mAb, termed SF1, that reacts with both human and mouse 6-sulfo sLe^x^. SF1 bound specifically to human and mouse HEVs and efficiently blocked lymphocyte homing to PLNs and nasal-associated lymphoid tissues (NALTs) in mice. We also showed that SF1 suppressed ovalbumin (OVA)-induced allergic rhinitis in mice, suggesting its potential for the functional analysis of 6-sulfo sLe^x^ under physiological and pathophysiological conditions. Hence, SF1 may serve as a novel therapeutic agent against immune-related diseases.

## Results

### Generation of mAb SF1 reactive with 6-sulfo sLe^x^

To obtain an anti-6-sulfo sLe^x^ mAb, GlcNAc6ST-1/-2 DKO mice were immunized with Chinese hamster ovary (CHO) cells stably expressing 6-sulfo sLe^x^ that had been transiently transfected with an expression vector encoding Cmah. Splenocytes of immunized mice were fused with mouse P3X63Ag8.653 myeloma cells to generate hybridomas. The culture supernatants were screened for immunoreactivity with the HEVs of WT mice and for the lack of immunoreactivity with the HEVs of GlcNAc6ST-1/-2 DKO^8^ and FucT-IV/-VII DKO^7^ mice lacking 6-sulfo sLe^x^. As a result, one hybridoma clone secreting anti-6-sulfo sLe^x^ mAb SF1 (mouse IgG_1_, κ) was established.

To compare the carbohydrate-binding specificity of mAb SF1 with that of previously established mAbs reactive with mouse HEVs, immunofluorescence staining of PLN frozen sections using biotinylated mAbs was performed (Fig. 1b). SF1 strongly bound to the HEVs of WT mice, but not to those of GlcNAc6ST-1/-2 DKO or FucT-IV/-VII DKO mice, indicating that SF1 requires both GlcNAc-6-*O*-sulfate and α1,3-fucose modifications. In contrast, the binding of mAbs S2^14^ and F2^16^ to HEVs was exclusively eliminated in GlcNAc6ST-1/-2 DKO and FucT-IV/-VII DKO mice, respectively, indicating that these mAbs require either sulfate or α1,3-fucose, but not both modifications. The staining of HEVs by SF1 was significantly diminished after neuraminidase treatment of the frozen sections, whereas that by mAb MECA-79^12^ was unaffected (Fig. 1c), indicating that SF1 also requires the sialylation of glycans. Collectively, these results indicate that SF1 binds to HEVs in mouse PLNs in a manner dependent on the sulfate, fucose, and sialic acid modifications.

### Glycan-binding specificity and dissociation constant of SF1

**Figure 2:**
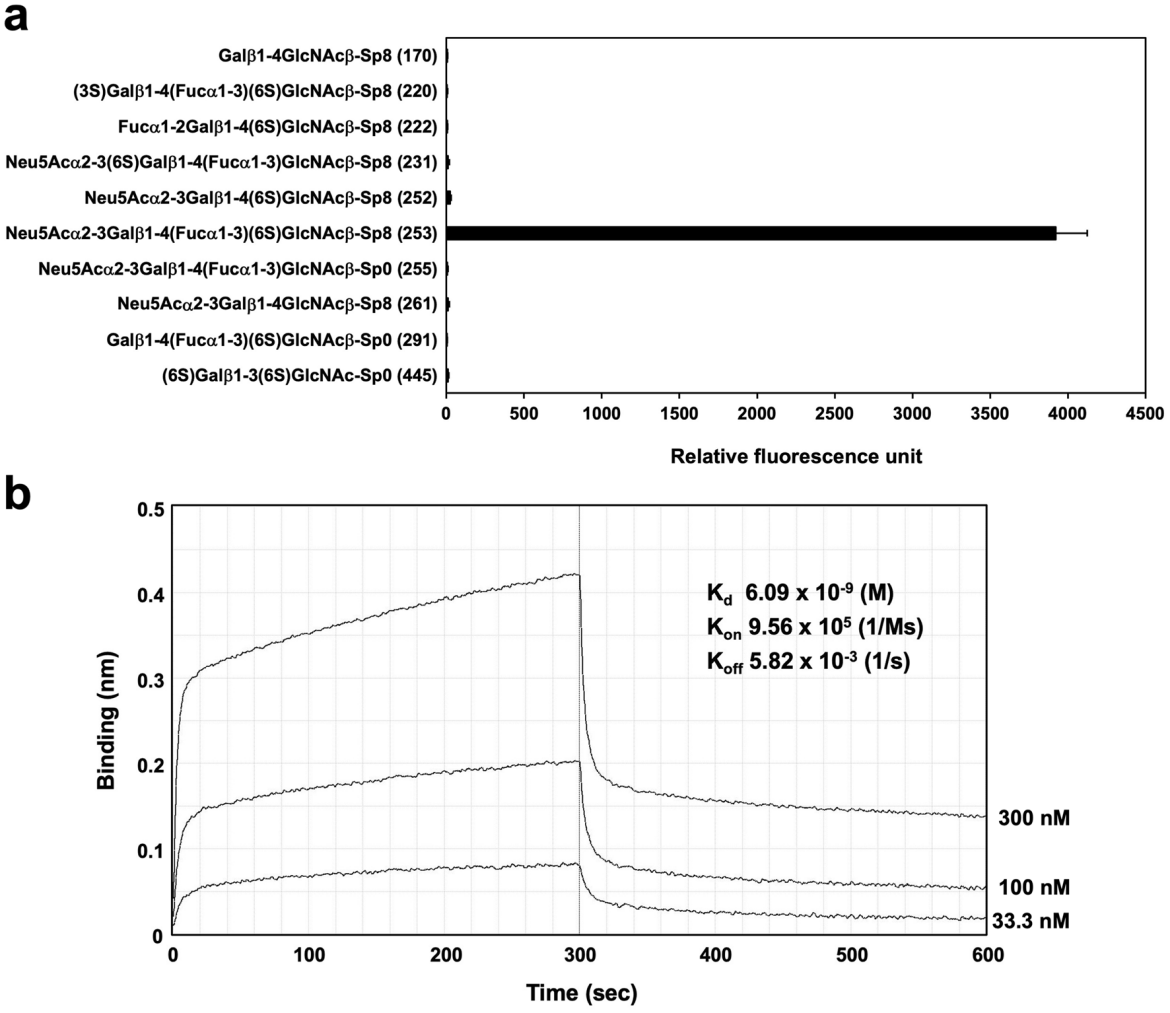
Glycan array analysis and biolayer interferometry of the binding between SF1 and 6-sulfo sLe^x^. (**a**) Glycan array of the Consortium for Functional Glycomics was probed with SF1. Error bars represent the mean ± standard deviation (SD) of four measurements. Glycan numbers are shown in parentheses after their structures. (**b**) Biolayer interferometry using Octet RED96. Binding of SF1 (33.3, 100, and 300 nM) was measured using streptavidin-coupled biosensor tips coated with biotinylated 6-sulfo sLe^x^.

To determine the glycan-binding specificity of SF1 in more detail, glycan array analysis was performed. SF1 specifically bound to 6-sulfo sLe^x^ (Glycan #253) among all glycans on the array (Supplementary Table S1). Figure 2a shows the results of 6-sulfo sLe^x^ and other representative glycans with related structures on the array. In contrast to mAbs S1 and S2^14^, SF1 did not bind to 6-sulfo sialyl LacNAc (Glycan #252), indicating that α1,3-fucose is required for binding. Similarly, in contrast to mAbs F1 and F2^16^, SF1 failed to bind to sLe^x^ (Glycan #255), indicating that the sulfate group is also required for binding. Consistent with the results of sialidase treatment described above, SF1 did not bind to 6-sulfo Lewis x (Glycan #291), lacking terminal α2,3-linked sialic acid. Substitution of sialic acid with sulfate at 3-OH of the galactose residue (Glycan #220) resulted in the complete loss of binding. In addition, SF1 did not bind to 6ʹ-sulfo sLe^x^ (Glycan #231), which was previously reported to interact with L-selectin^19^, or α1-2 fucosylated 6-sulfo LacNAc (Glycan #222), which is present in the *O*-glycans of murine colonic mucins^20^. Consistently, SF1 did not bind to LacNAc (Glycan #170), sialyl LacNAc (Glycan #261), and disulfated LacNAc (Glycan #445). Collectively, these results indicate that SF1 is highly specific to 6-sulfo sLe^x^ glycan.

To determine the dissociation constant (K_d_) of the binding between SF1 and 6-sulfo sLe^x^, we performed biolayer interferometry using Octet RED96 (Fig. 2b). Using streptavidin-coupled biosensor tips that had been pre-incubated with biotinylated 6-sulfo sLe^x^, the association rate constant (K_on_) and dissociation rate constant (K_off_) were determined to be 9.56 × 10^5^ (1/Ms) and 5.82 × 10^–3^ (1/s), respectively. We did not detect any binding of SF1 to biotinylated sialyl LacNAc, confirming that the specificity of the binding of SF1 to 6-sulfo sLe^x^. K_d_ was calculated as K_off_/K_on_. The K_d_ value of the binding between SF1 and 6-sulfo sLe^x^ was calculated to be 6.09 × 10^–9^ (M).

### Inhibitory effects of SF1 on L-selectin-Fc binding to HEVs and lymphocyte rolling

**Figure 3:**
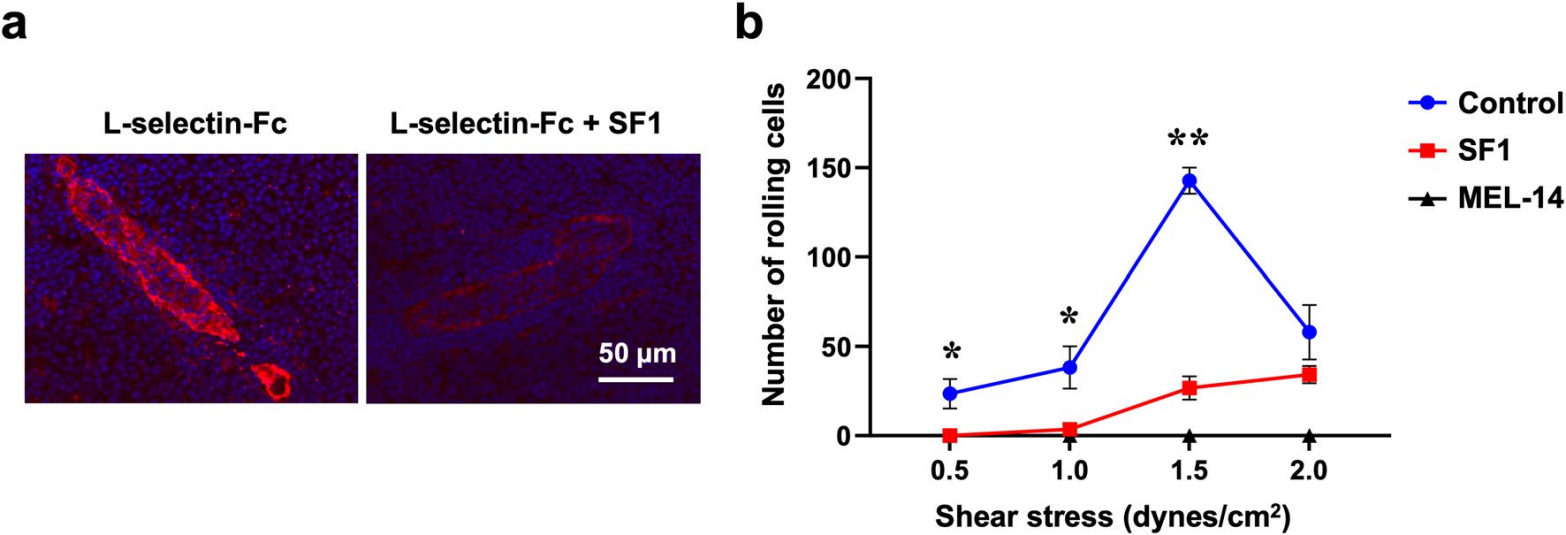
Effects of SF1 on the binding of L-selectin-Fc to HEVs in PLN sections and L-selectin-mediated lymphocyte rolling. (**a**) Binding of L-selectin-Fc to PLN HEVs in C57BL/6 WT mice in the presence (+ *SF1*) or absence of SF1. (**b**) The number of rolling cells was counted for 60 s at shear stresses of 0.5, 1.0, 1.5, and 2.0 dynes/cm^2^. Lymphocytes from mouse spleens that had been pretreated with or without MEL-14 were introduced into the flow chamber with the bottom surface covered with a monolayer of CHO/CD34/C1/C2/FucT-7/GlcNAc6ST-2 cells that had been pretreated with or without SF1. Error bars represent the standard error of the four measurements. *P < 0.05, **P < 0.01 (SF1 vs. Control).

Since 6-sulfo sLe^x^ functions as a major ligand for L-selectin, we examined the blocking effects of SF1 on the binding of L-selectin to its ligands on HEVs. To this end, frozen sections of mouse PLNs were incubated with the L-selectin-Fc fusion protein in the presence or absence of SF1, followed by incubation with biotinylated anti-human IgG secondary antibody and Alexa Fluor 594-labeled streptavidin (Fig. 3a). SF1 strongly inhibited the binding of L-selectin-Fc to HEVs, indicating that SF1 inhibits the interaction between the lymphocyte homing receptor, L-selectin, and its ligands expressed on HEVs.

To determine whether SF1 blocks L-selectin-mediated lymphocyte rolling under physiological flow conditions, a parallel-plate flow chamber assay was performed. As shown in Fig. 3b, CHO cells expressing CD34 core proteins and 6-sulfo sLe^x^ glycans supported the L-selectin-dependent rolling of mouse lymphocytes, which was completely blocked by the anti-L-selectin mAb MEL-14^21^ under physiological shear stress. As a result, the rolling was significantly inhibited by SF1, indicating that SF1 has the ability to block the interaction between L-selectin and its ligands under physiological shear stress.

### Binding of SF1 to L-selectin ligand glycoproteins and its blocking effects on lymphocyte homing to PLNs

**Figure 4:**
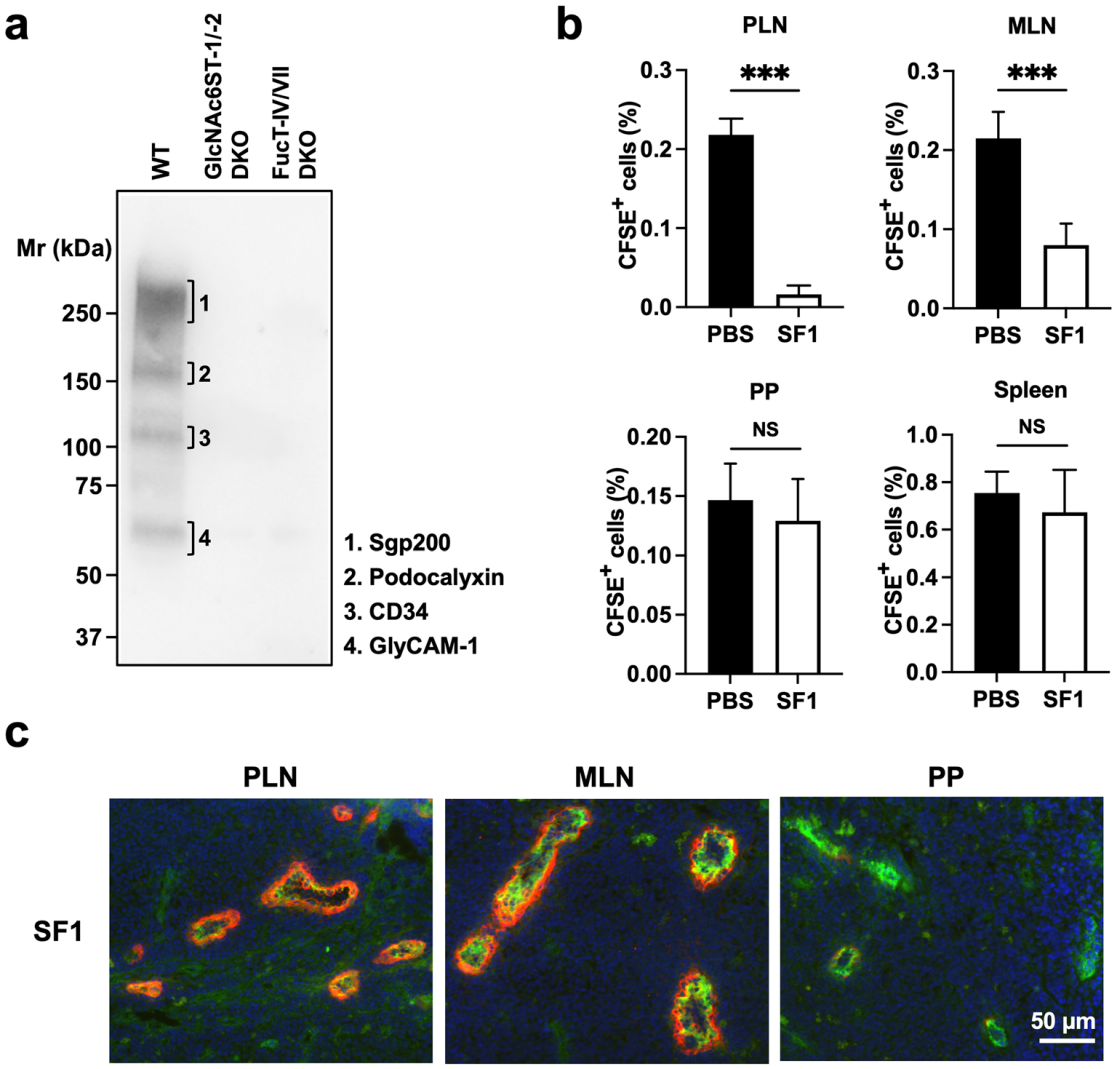
SF1 specifically binds to four glycoproteins and inhibits lymphocyte homing to PLNs and MLNs in C57BL/6 mice. (**a**) Western blotting. The blots of PLN lysates from C57BL/6 WT, GlcNAc6ST-1/-2 DKO, or FucT-IV/-VII DKO mice were probed with SF1, followed by HRP-conjugated anti-mouse IgG that specifically reacts with undenatured mouse IgG. (**b**) Inhibition of lymphocyte homing by SF1 in C57BL/6 mice. CFSE-labeled lymphocytes from C57BL/6 WT mice were injected into the tail veins of the same strain of WT mice that had been pre-injected with SF1 or PBS 2 h before the injection of labeled lymphocytes. Lymphocyte homing to PLNs (containing CLNs, BLNs, and ILNs), MLNs, PPs, and spleen is shown as the percentage of CFSE^+^ lymphocytes among the total lymphocytes in each lymphoid organ (n = 5). Each bar represents the mean ± SD. ***P < 0.001; *NS* not significant. (**c**) Immunofluorescence: Frozen sections of PLNs, MLNs, and PPs from C57BL/6 WT mice were incubated with biotinylated SF1 and Alexa Fluor 488-conjugated anti-mouse CD31 (*green*), streptavidin-Alexa Fluor 594 (*red*), and DAPI (*blue*). *Bar*, 50 µm.

L-selectin interacts with a unique set of glycoproteins, termed peripheral node addressins^22^, such as Sgp200^23^, podocalyxin-like protein^24^, CD34^25^, and glycosylation-dependent cell adhesion molecule-1 (GlyCAM-1)^26^. To determine whether SF1 binds to these glycoproteins, we performed western blotting analysis using tissue lysates of PLNs from WT, GlcNAc6ST-1/-2 DKO, and FucT-IV/-VII DKO mice (Fig. 4a, Supplementary Fig. S1). Consistent with the ability of SF1 to block the binding of L-selectin to its ligands on HEVs, SF1 mainly bound to four glycoproteins corresponding to the molecular sizes of the peripheral node addressins in WT mice, but not those from GlcNAc6ST-1/-2 or FucT-IV/-VII DKO mice lacking 6-sulfo sLe^x^.

To assess the functional effects of SF1 in vivo, a lymphocyte homing assay using C57BL/6 WT mice was performed. As shown in Fig. 4b, SF1 inhibited lymphocyte homing to PLNs (containing cervical lymph nodes [CLNs], brachial lymph nodes [BLNs], and inguinal lymph nodes [ILNs]) by more than 90%. Homing to the mesenteric lymph nodes (MLNs) was also suppressed by approximately 60%. In contrast, SF1 did not show any inhibitory effects on lymphocyte homing to Peyer's patches (PPs) and spleen. Consistent with these findings, SF1 clearly bound to HEVs in PLNs and MLNs, but minimally bound to those in PPs (Fig. 4c).

### Suppressive effects of SF1 on lymphocyte homing to NALTs and a murine allergic rhinitis model

**Figure 5:**
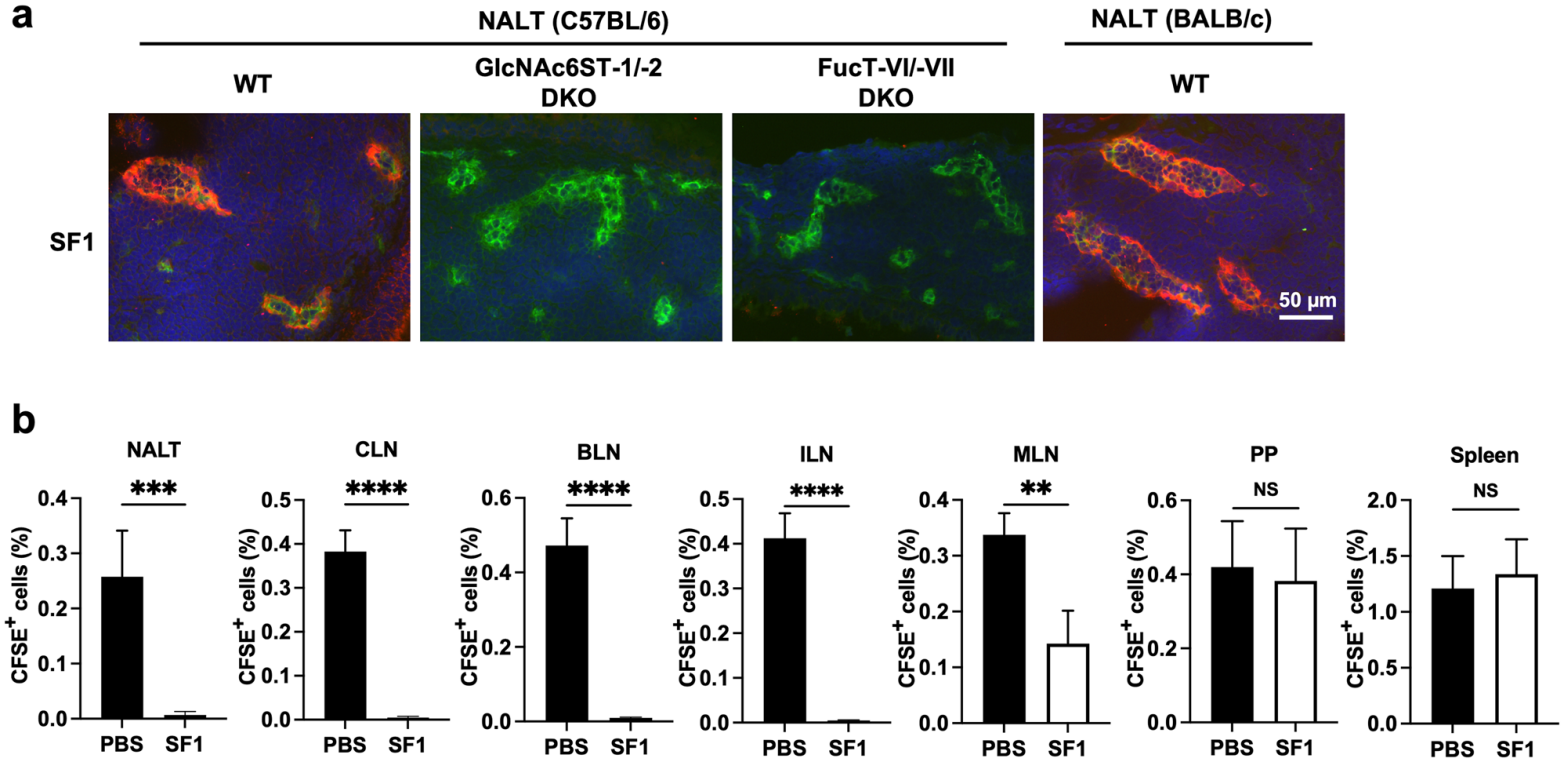
Binding of SF1 to NALT HEVs and its effect on lymphocyte homing in BALB/c mice. (**a**) Immunofluorescence: Frozen sections of NALTs from C57BL/6 WT, GlcNAc6ST-1/-2 DKO, FucT-IV/-VII DKO, and BALB/c WT mice were incubated with biotinylated SF1 and Alexa Fluor 488-conjugated anti-mouse CD31 (*green*), streptavidin-Alexa Fluor 594 (*red*), and DAPI (*blue*). *Bar*, 50 µm. (**b**) Inhibition of lymphocyte homing by SF1 in BALB/c mice. CFSE-labeled lymphocytes from BALB/c WT mice were injected into the tail veins of the same strain of WT mice that had been pre-injected with SF1 or PBS 2 h before the injection of labeled lymphocytes. Lymphocyte homing is shown as the percentage of CFSE^+^ lymphocytes among the total lymphocytes in each lymphoid organ. Each bar represents the mean ± SD (n = 4). **P < 0.01; ***P < 0.001; ****P < 0.0001; *NS* not significant.

NALT is a secondary lymphoid organ in mice, where immune responses against inhaled allergens primarily occur^27^. To determine the effects of SF1 in a murine allergic rhinitis model, we examined the reactivity of SF1 to HEVs in NALTs using immunofluorescence. As shown in Fig. 5a, HEVs in NALTs of C57BL/6 mice were reactive with SF1, which was eliminated in GlcNAc6ST-1/-2 and FucT-IV/-VII DKO mice. In addition, HEVs in the NALTs of BALB/c mice were also strongly reactive with SF1. Because BALB/c mice are T helper 2 (Th2)-prone and are often used for murine models of allergy^28^, we used this mouse strain for further analysis of lymphocyte homing and OVA-induced allergic rhinitis model. In the homing assay using BALB/c mice, each secondary lymphoid organ was collected separately (Fig. 5b). Similar to what was observed in C57BL/6 mice, lymphocyte homing to PLNs (CLNs, BLNs, and ILNs) was strongly blocked by SF1 by > 95%, whereas that to MLNs was partially blocked by 60%, and that to PPs and spleen was unaffected by SF1. Consistent with the immunofluorescence data, SF1 significantly inhibited lymphocyte homing to NALTs by > 95%.

**Figure 6:**
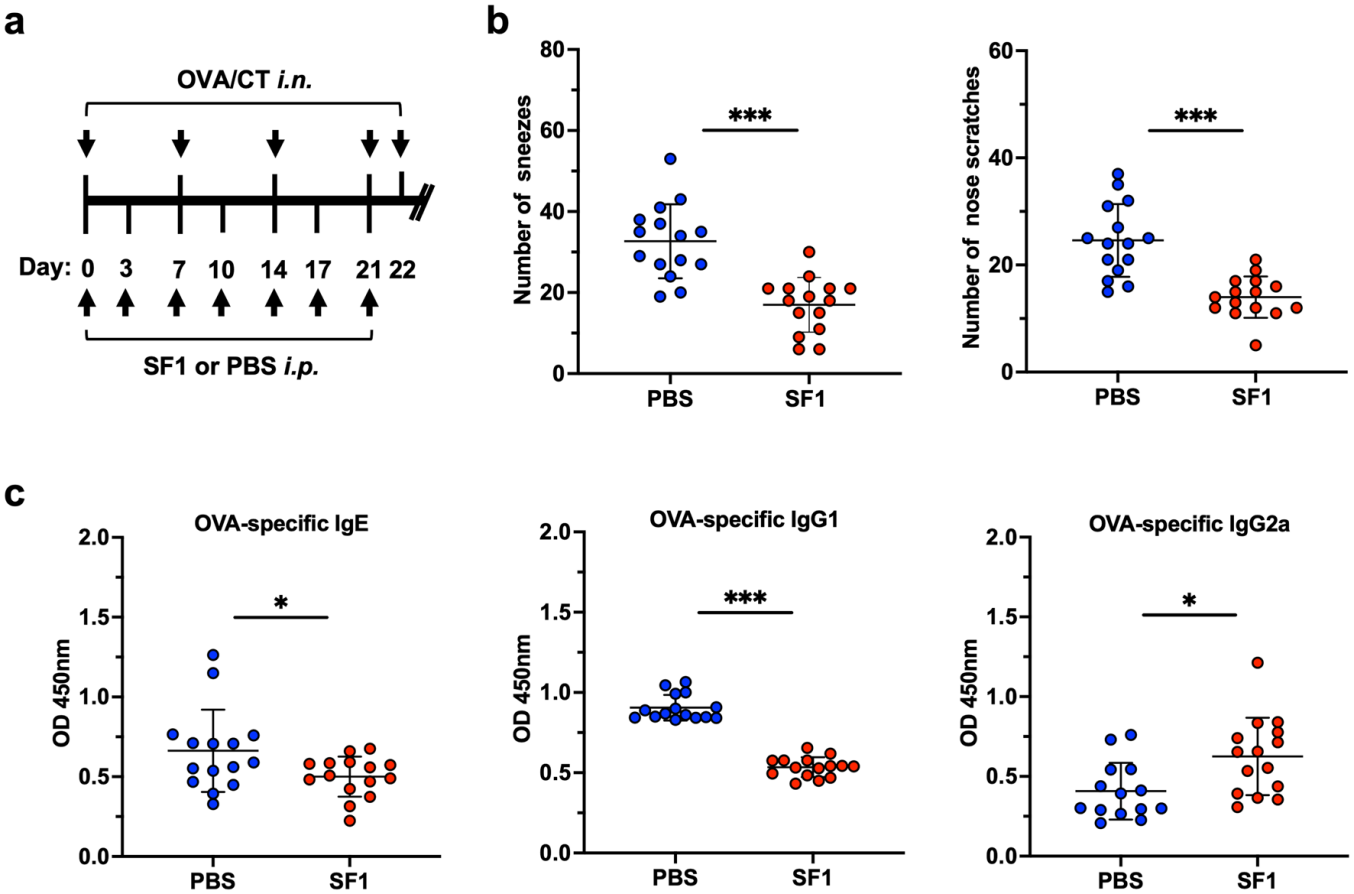
Effects of SF1 on OVA-induced allergic rhinitis in BALB/c mice. (**a**) Scheme of the OVA-induced allergic rhinitis model and SF1 administration in BALB/c mice. BALB/c WT mice were immunized intranasally (*i.n.*) with OVA and CT on days 0, 7, 14, and 21. SF1 or PBS was administered intraperitoneally (*i.p.*) on days 0, 3, 7, 10, 14, 17, and 21. On day 22, the mice received the final intranasal administration of a mixture of OVA and CT. (**b**) Clinical symptoms of allergic rhinitis. Immediately after the final intranasal administration of OVA and CT on day 22, the frequency of sneezing and nasal rubbing motion was measured for 12 min. (**c**) OVA-specific IgE, IgG1, and IgG2a levels. Sera were collected on day 22 after the final antigen administration, and ELISA was performed to detect OVA-specific antibodies. Each bar represents the mean ± SD (n = 14–15). *P < 0.05; ***P < 0.001.

To determine the effects of SF1 on allergic rhinitis in mice, we induced allergic rhinitis in BALB/c mice via the intranasal administration of OVA in the presence or absence of SF1 (Fig. 6a) and examined their clinical symptoms. The number of sneezes and nose scratches after the final intranasal immunization with OVA was significantly diminished in SF1-administered mice (Fig. 6b). Consistently, the levels of OVA-specific serum IgE, a key immunoglobulin that causes allergic symptoms, were diminished in SF1-administered mice (Fig. 6c). The levels of OVA-specific IgG1 produced by Th2 immune responses, similar to IgE, were also reduced, whereas those of OVA-specific IgG2a produced by Th1 immune responses were increased, suggesting that Th2 immune responses are specifically blocked in OVA-induced allergic rhinitis.

**Figure 7:**
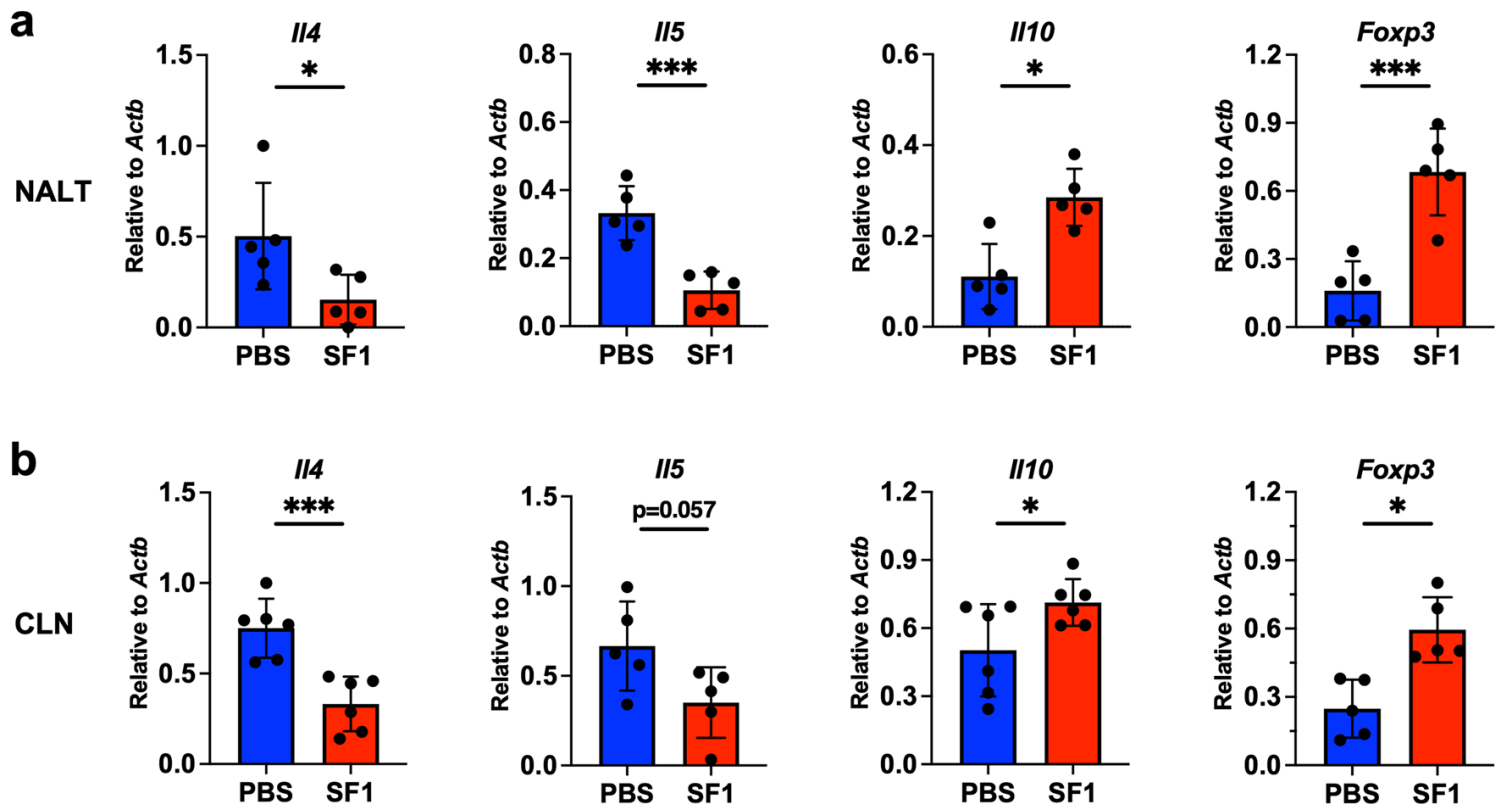
Effects of SF1 on the expression levels of cytokines and Foxp3 in allergic rhinitis-induced BALB/c mice. RT-qPCR analysis of the genes encoding IL-4 (*Il4*), IL-5 (*Il5*), IL-10 (*Il10*), and Foxp3 (*Foxp3*) was performed using total RNA samples from NALTs (**a**) and CLNs (**b**) collected 22 days after the final administration of OVA and CT, as shown in Fig. 6a. The expression of each gene was normalized to that of β-actin (*Actb*) using the ∆∆Ct method. Each bar represents the mean ± SD (n = 5–6). *P < 0.05; ***P < 0.001.

To determine the molecular basis of this suppression, we performed reverse transcription-quantitative PCR (RT-qPCR) analysis using total RNAs from NALTs and CLNs, where intranasally administered antigens were accumulated (Fig. 7a,b). The expression levels of Th2 cytokine, interleukin (IL)-4, which is critical for IgE class switching^29^, were significantly reduced by SF1 administration in both NALTs and CLNs. The levels of another Th2 cytokine, IL-5, were also significantly reduced by SF1 in NALTs, although the reduction in CLN levels was not statistically significant. In contrast, the expression levels of the immunosuppressive cytokine IL-10, which is secreted from regulatory T (Treg) cells^30^ were increased by SF1 administration in both NALTs and CLNs. Consistently, the expression levels of forkhead box P3 (Foxp3), a master transcription factor critical for the development and maintenance of Treg cells^31^, was increased by SF1 administration.

### Immunohistochemical staining of normal human tissues with SF1

**Figure 8:**
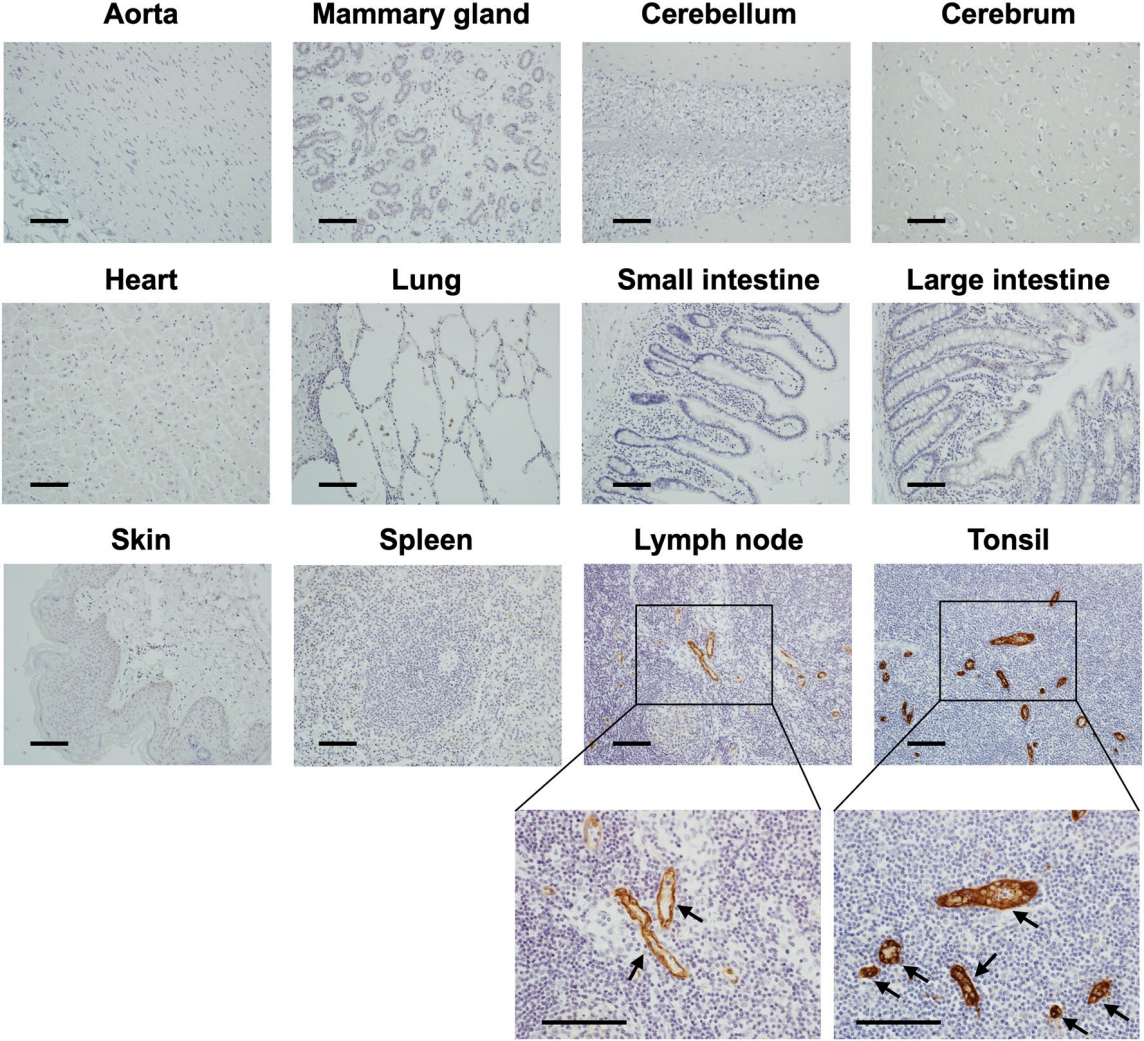
Immunohistochemical staining of paraffin-embedded normal human tissues with SF1. The SF1 antibody showed no binding in the aorta, breast, cerebellum, cerebrum, heart, lung, small intestine, large intestine, skin, or spleen, whereas it selectively bound to HEVs in the lymph nodes and tonsils of normal human tissues. *Arrows* in the enlarged images of the lymph nodes and tonsils indicate the HEVs. *Bars*, 100 µm.

To confirm the tissue-binding specificity of SF1, immunohistochemical staining of normal human tissues with SF1 was performed. As shown in Fig. 8, SF1 showed no staining in the aorta, mammary gland, cerebellum, cerebrum, heart, lung, small intestine, large intestine, skin, and spleen, but selectively stained the lymph nodes and tonsils. From the enlarged photographs, it was confirmed that SF1 specifically binds to HEVs in these lymphoid organs. Because human lymphocytes are selectively recruited to these lymphoid organs via HEVs to evoke immune responses, these results, together with the abovementioned effects of SF1 on lymphocyte homing and the murine allergic rhinitis model, suggest that SF1 may be useful as a therapeutic agent against immune-related diseases in humans.

## Discussion

In the present study, we established a novel mAb against 6-sulfo sLe^x^, termed SF1, using a previously established method to generate anti-glycan mAbs by immunizing mice deficient in glycan-synthesizing enzymes with transfectants that overexpress the missing enzymes^14,18^. Here, we provide biochemical evidence of the specificity and affinity by glycan array and biolayer interferometry analyses. We also provide evidence that SF1 selectively binds to L-selectin ligand glycoproteins and inhibits L-selectin-mediated rolling. In vivo functional analyses indicated that SF1 significantly inhibited lymphocyte homing to PLNs and NALTs, and allergic rhinitis in mice. Together with the results of histochemical analysis of human tissue sections using SF1, the results of this study suggest that 6-sulfo sLe^x^ can serve as a therapeutic target against immune-related diseases.

Thus far, mAb MECA-79^12^ has frequently been used as a marker for HEVs. However, MECA-79 recognizes the sulfated extended core 1 structure, and its epitope does not completely match with that recognized by L-selectin containing sulfate, fucose, and sialic acid. Previously established mAbs, including S1, S2, F1, F2, and CL41, all recognize a portion of the 6-sulfo sLe^x^ structure^14–16^, although they can bind both to human and mouse HEVs. Another mAb, G152, binds to the 6-sulfo sLe^x^ structure^17^; however, because of its lack of reactivity to HEVs in mice, it cannot be used to assess the in vivo function of this glycan epitope in mouse models. In contrast to these previously established mAbs, SF1 is highly specific to the 6-sulfo sLe^x^ structure expressed in both humans and mice, as assessed using immunofluorescence and glycan array analyses.

In our western blotting analysis, SF1 preferentially bound to glycoprotein species of > 200, 170, 100, and 60 kDa, which were probably Sgp200^23^, podocalyxin-like protein^24^, CD34^25^, and GlyCAM-1^26^, respectively. Although previous studies have revealed that these glycoproteins are modified with MECA-79-reactive glycans^22,23^, it is unclear whether they can be modified with 6-sulfo sLe^x^, except for GlyCAM-1, whose detailed glycan structural analysis has been performed^8^. The use of SF1 in this study suggests that these glycoproteins are indeed modified with 6-sulfo sLe^x^, although their precise core protein and glycan structures remain elusive because of the small quantities available from the murine lymph nodes.

Notably, SF1 is an IgG1 class antibody with a K_d_ of 6.09 × 10^–9^ M toward 6-sulfo sLe^x^ glycan. Because L-selectin is known to bind to one of its glycoprotein ligands, GlyCAM-1, with a K_d_ of 1.08 × 10^–4^ M^32^, the affinity of SF1 toward 6-sulfo sLe^x^ glycan may be high enough to block L-selectin and its ligand interaction. Consistently, our immunofluorescence study using L-selectin-Fc and ex vivo rolling assays showed that SF1 inhibited L-selectin and its ligand interaction. Furthermore, SF1 strongly inhibited lymphocyte homing to PLNs by more than 90% in both C57BL/6 and BALB/c mice, which is known to be mediated by the interaction between L-selectin and its ligands^33^. In contrast, previous studies using GlcNAc6ST-1/-2 DKO mice indicated that lymphocyte homing to PLNs in these mutant mice was diminished by 75% and the remaining homing activity appeared to be mediated by unsulfated sLe^x^^8,9^. One possible explanation for the greater suppressive activity of SF1 on lymphocyte homing than that observed in DKO mice is that SF1 may also block the weak interaction between L-selectin and unsulfated sLe^x^ by steric hindrance. Another possibility is that unsulfated sLe^x^ may support significant levels of lymphocyte homing only when it is overexpressed in the absence of GlcNAc6ST-1 and -2, as evidenced by the glycan structural study of GlyCAM-1 in DKO mice^8^. These possibilities are not mutually exclusive and both may be involved. In either case, the strong blocking ability of SF1 on lymphocyte homing to PLNs warrants its possible use as an immunomodulatory agent against immune-related diseases.

SF1 did not show any inhibition of lymphocyte homing to PPs, most likely because it is mainly mediated by the interaction between α4β7 integrin and mucosal addressin cell adhesion molecule-1 (MAdCAM-1)^34^ and also because SF1 bound only very weakly (less than 5%) to HEVs in PPs. The partial inhibition of SF1 on lymphocyte homing to MLNs is probably because it is mediated by both L-selectin and its ligand interaction and α4β7 integrin and MAdCAM-1 interaction^35^. NALT is a mucosal lymphoid tissue in rodents that is equivalent to Waldeyer's ring in humans, including the tonsils and adenoids^27^. Consistent with our previous study using GlcNAc6ST-1/-2 DKO mice^36^, SF1 significantly blocked lymphocyte homing to NALT by more than 95%, although PPs and NALT are the two main constituents of mucosa-associated lymphoid tissue (MALT) and are important inductive sites for mucosal immunity against ingested and inhaled antigens, respectively^37^. Since SF1 is strongly reactive with human tonsils, it may suppress both mouse and human nasal allergic responses without affecting the immune responses against ingested antigens in PPs.

In a murine allergic rhinitis model, SF1 suppressed symptoms, such as sneezing and nose scratching, after nasal administration of the antigen in association with the reduction of antigen-specific IgE that causes allergic symptoms. SF1 also suppressed the expression of the Th2 cytokine, IL-4, which is critical for IgE class switching in B cells^38^. These results suggest that the blockage of lymphocyte homing to NALTs and CLNs by SF1 reduced the chance of lymphocytes encountering their cognate antigens, leading to diminished Th2-cell differentiation in these lymphoid organs. Another observation is that the expression levels of the immunosuppressive cytokine, IL-10, and Treg-specific transcription factor, Foxp3^31^, were increased in NALTs and CLNs in SF1-administered mice, consistent with our previous study using GlcNAc6ST-1/-2 DKO mice^36^. We previously reported that the homing of Treg cells to NALT is less dependent on L-selectin and its ligand interaction and more dependent on P-selectin glycoprotein ligand 1–P-selectin and CD44–hyaluronic acid interactions compared to that of conventional T cells^1,36^. Therefore, we speculated that homing of Treg cells may have been less affected by SF1, which may be involved, at least partly, in the suppression of Th2 responses by SF1 in these lymphoid organs.

Because SF1 binds not only to mouse, but also to human tissues, it can be useful for the histopathological staining of human clinical samples. In particular, it will be interesting to examine the staining of SF1 using specimens of immune-related diseases in which HEV-like blood vessels are ectopically induced at the sites of chronic inflammation^3,39–42^ including rheumatoid arthritis^43^, Sjögren syndrome^44^, and ulcerative colitis^45^. Because SF1 strongly blocks L-selectin-mediated lymphocyte homing and recruitment, determination of the expression of 6-sulfo sLe^x^ in HEV-like blood vessels at sites of chronic inflammation in clinical samples may lead to a novel therapeutic intervention against these diseases in the future.

In conclusion, we developed a novel anti-6-sulfo sLe^x^ mAb SF1 that reacts with both human and mouse tissues. Since SF1 significantly blocks lymphocyte homing and allergic rhinitis in mice, future studies using SF1 will advance our understanding of the role of 6-sulfo sLe^x^ in health and disease.

## Methods

### Animals

Animal study is approved by the Animal Research Committee of Chiba University. GlcNAc6ST-1/-2 DKO^14^ and FucT-IV/-VII DKO mice^7^ were maintained as previously described. C57BL/6 WT, BALB/c, and BALB/c nu/nu mice were purchased from Charles River Laboratories (Yokohama, Japan). Mice were treated in accordance with the guidelines of the Animal Research Committee of Chiba University. The reporting in the manuscript follows the recommendations in the ARRIVE (Animals in Research: Report-ing In Vivo Experiments) guidelines^46^.

### Generation of mAb SF1

CHO cells stably expressing human CD34, human Core1 β1,3-*N*-acetylglucosaminyl transferase (GlcNAcT), human Core2 β1,6-GlcNAcT-I, human FucT-VII, and mouse GlcNAc6ST-2 (CHO/CD34/C1/C2/FucT-7/GlcNAc6ST-2 cells) were transiently transfected with mouse Cmah using the FuGENE 6 transfection reagent (Promega, Madison, WI, USA). After 48 h, the cells were suspended in PBS and mixed with Imject Alum (Thermo Fisher Scientific, Waltham, MA, USA) in 1:1 ratio. The mixture was injected intraperitoneally into GlcNAc6ST-1/-2 DKO mice three times at 2-week intervals. Four days after the final immunization, lymphocytes from the spleens of immunized mice were fused with P3X63Ag8.653 myeloma cells (American Type Culture Collection, Manassas, VA, USA) using PEG solution (MW 1450; Sigma-Aldrich, St. Louis, MO, USA). Hybridomas were selected in medium containing HAT supplement (Thermo Fisher Scientific). Supernatants reactive with the HEVs of WT mice, but not with those of GlcNAc6ST-1/-2 DKO and FucT-IV and -VII DKO mice, were selected via immunofluorescence using a mixture of Alexa Fluor 488-conjugated anti-mouse IgG (Thermo Fisher Scientific) and Alexa Fluor 594-conjugated anti-mouse IgM (Thermo Fisher Scientific) as secondary antibodies. Hybridomas that secreted mAbs with the desired specificity were cloned using limiting dilution. The isotype of the mAb SF1 thus obtained was IgG1 (k), as determined using an isotyping kit (GE Healthcare, Chicago, IL, USA). Hybridomas secreting SF1 were expanded and injected into BALB/c nude mice (5.0 × 10^6^ cells/mouse) that had been pre-injected with 500 μL/mouse of pristane (2,6,10,14-tetramethyl pentadecane; Sigma-Aldrich) a few weeks before the cell injection, and the ascitic fluid was collected. SF1 was then purified from ascitic fluid using a caproic acid (6-aminohexanoic acid; FUJIFILM Wako, Osaka, Japan) precipitation method. In certain cases, purified SF1 was biotinylated with EZ-Link Sulfo-NHS-LC-biotin (Thermo Fisher Scientific), according to the manufacturer’s protocol.

### Immunofluorescence

Acetone-fixed, frozen Sections (7-μm) from mice that had been treated with or without 5,000 U/mL α2-3,6,8 neuraminidase (NEW ENGLAND BioLabs, Inc., Ipswich, MA, USA) for 5 h at 37 °C were incubated with PBS containing 3% BSA (Sigma-Aldrich) to block non-specific binding sites, followed by incubation with 5 μg/mL biotinylated SF1, 1 μg/mL biotinylated F2, 5 μg/mL biotinylated S2, or 5 μg/mL biotinylated MECA-79 (BioLegend, San Diego, CA, USA) together with or without 2 μg/mL Alexa Fluor 488-conjugated anti-mouse CD31 (MEC13.3, BioLegend). After washing, the sections were incubated with 0.5 μg/mL Alexa Fluor 594-conjugated streptavidin (Thermo Fisher Scientific) and 0.5 μg/mL 4ʹ,6-diamidino-2-phenylindole dihydrochloride (DAPI; Thermo Fisher Scientific), and mounted using Fluoromount (Diagnostic BioSystems, Pleasanton, CA, USA). For immunofluorescence using recombinant human L-selectin-Fc chimera protein (R&D Systems, Inc., Minneapolis, MN, USA), the tissue sections were incubated with a mixture of 5 μg/mL human L-selectin-Fc fusion protein and 40 μg/mL SF1 in Buffer A (20 mM HEPES–NaOH, 150 mM NaCl, 1 mM MgCl_2_, 1 mM CaCl_2_, pH 7.4, containing 0.1% BSA). After overnight incubation at 4 °C, the sections were washed with Buffer A and incubated with 1 μg/mL biotinylated goat anti-human IgG F(ab’)_2_ with minimum cross-reactivity with bovine, horse, mouse, and rat serum proteins (Rockland Immunochemicals, Inc., Limerick, PA, USA) for 2 h at room temperature. After washing with Buffer A, the sections were incubated with 1 μg/mL Alexa Fluor 594-conjugated streptavidin (Thermo Fisher Scientific) and 0.5 μg/mL DAPI for 1 h at room temperature. All images were obtained using a fluorescence microscope (BZ-9000; KEYENCE, Osaka, Japan).

### Glycan array analysis

The purified SF1 antibody was subjected to glycan array analysis using microarray slides at the Consortium for Functional Glycomics (Core H), as described previously^14,16^. Briefly, SF1 was dialyzed against 20 mM Tris–HCl (pH 7.4), containing 150 mM NaCl. The dialyzed antibody was diluted in 20 mM Tris–HCl (pH 7.4) containing 1% BSA, 0.05% Tween 20, 150 mM NaCl, 2 mM CaCl_2_, and 2 mM MgCl_2_ and applied to a glycan array. After incubation, the array was incubated with FITC-labeled anti-mouse IgG antibody and its binding was detected using a fluorescence array scanner.

### Biolayer interferometry

Biolayer interferometry was performed using Octet RED96 (ForteBio, Fremont, CA, USA), according to the manufacturer’s protocol. Briefly, streptavidin-coupled biosensor tips were coated with 2 µM biotinylated 6-sulfo sLe^x^ (6(GlcNAc)-HSO_3_SiaLe^x^-sp-biotin, GlycoTech Co., Gaithersburg, MD, USA). As a control, 2 µM biotinylated sialyl LacNAc (Neu5Acα2-3Galβ1-4GlcNAcβ-sp-biotin, GlycoTech Co.) was used instead of biotinylated 6-sulfo sLe^x^. After washing with PBS, purified SF1 at concentrations of 33.3, 100, and 300 nM was used to measure the K_on_ and K_off_ values of the binding between SF1 and 6-sulfo sLe^x^. The K_d_ value was determined as K_off_/K_on_.

### Rolling assay

CHO/CD34/C1/C2/FucT-7/GlcNAc6ST-2 cells stably expressing 6-sulfo sLe^x^ were cultured as monolayers in 35-mm culture dishes (Corning Inc., Corning, NY, USA). Freshly prepared mouse splenocytes were fluorescently labeled with 1 μM carboxyfluorescein diacetate succinimidyl ester (CFSE; Thermo Fisher Scientific) at 37 °C for 20 min and incubated with or without 10 μg/mL of purified MEL-14 (BioLegend) for 10 min at room temperature. At the same time, the dish-attached CHO/CD34/C1/C2/FucT-7/GlcNAc6ST-2 cells were incubated with or without 10 μg/mL purified SF1 for 10 min at room temperature. After incubation, the dishes were equipped with a parallel plate flow chamber (GlycoTech Co.), according to the manufacturer’s instructions. CFSE-labeled splenocytes were resuspended in buffer A at a density of 5 × 10^5^ cells/mL and introduced into the flow chamber at wall shear stresses of 0.5, 1.0, 1.5, and 2.0 dynes/cm^2^ using a syringe pump (Model 11 Plus, Harvard Apparatus Co., Holliston, MA, USA). Images were captured using an OptiMOS Scientific CMOS camera (Teledyne Photometrics, Tucson, AZ, USA) equipped with an inverted microscope (Axiovert S100, Carl Zeiss Meditec AG, Oberkochen, Germany) and analyzed using the Fiji ImageJ software.

### Western blotting

PLNs from C57BL/6 WT, GlcNAc6ST-1/-2 DKO, and FucT-IV/-VII DKO mice were mashed in ice-cold PBS containing 1% Triton X-100 and Halt Protease inhibitor cocktail (Thermo Fisher Scientific) and solubilized for 4 h with rotation at 4 °C. Lysates were centrifuged at 15,000 rpm for 10 min. The supernatants were collected and protein concentrations were determined using a BCA Protein Assay Kit (Thermo Fisher Scientific). The obtained lysates were stored at – 80 °C until use. Equal amount of lysate was applied to each lane of the SDS-PAGE gel, and western blotting was performed using 2 μg/mL purified SF1 and 1:1000 diluted horseradish peroxidase (HRP)-conjugated anti-mouse IgG (GeneTex, Irvine, CA, USA) that specifically reacts with undenatured mouse IgG. After incubating the membrane with Clarity Western ECL Substrate (Bio-Rad), bands were detected using the FUSION FX7.EDGE Imaging system (Vilber Lourmat, Collègien, France).

### Lymphocyte homing assay

The lymphocyte homing assay was performed as described previously with some modifications^8,14,16^. Briefly, MLN lymphocytes and splenocytes from C57BL/6 or BALB/c WT mice were labeled with 1 µM CFSE. After washing, 1.5 × 10^7^ labeled cells in 100 µL of sterile PBS were injected intravenously into recipient WT mice that had been pre-injected intravenously with 200 µL of 0.5 mg/mL purified SF1 mAb (100 μg per mouse, dissolved in sterile PBS) or sterile PBS 2 h before the injection of labeled lymphocytes. Two hours after the injection of the cells, the number of CFSE^+^ cells in the cell suspensions prepared from the recipient secondary lymphoid organs was determined using flow cytometry. The cells were analyzed via flow cytometry using a CytoFLEX flow cytometer (Beckman Coulter, Brea, CA, USA).

### Intranasal immunization

BALB/c mice were anesthetized with isoflurane (Pfizer Inc., New York, NY, USA) and immunized intranasally on days 0, 7, 14, and 21 with 100 µg of OVA (Sigma-Aldrich) in PBS together with 0.5 µg of cholera toxin (CT; List Biological Laboratories Inc., Campbell, CA, USA), which is used as a mucosal adjuvant in a total volume of 13 µL per mouse. Mice were injected intraperitoneally with 200 μL of 0.5 mg/mL purified SF1 mAb (100 μg per mouse, dissolved in sterile PBS) or sterile PBS on days 0, 3, 7, 10, 14, 17, and 21. On day 22, mice received the final intranasal administration of a mixture of OVA and CT, as described above, and the frequency of sneezing and nasal rubbing motions were counted for 12 min. Thereafter, serum was collected from each mouse and stored at – 80 °C until use for the measurement of OVA-specific antibodies. In addition, NALTs and CLNs were collected on day 22, immersed in RNAlater solution (Thermo Fisher Scientific), and stored at – 80 °C until use for RT-qPCR.

### ELISA for the measurement of serum antibody levels

To detect OVA-specific IgE in mouse serum, wells of a 96-well ELISA plate (Costar Assay Plate, half area; CORNING) were coated overnight with 2.0 μg/mL anti-mouse IgE (BioLegend) and the non-specific binding sites were blocked with 3% BSA in PBS for 2 h. Diluted serum samples (1:4 dilution) were then added to the wells and incubated for 2 h. After washing with 0.05% Tween 20 in PBS, 5.0 µg/mL biotinylated-OVA labeled with EZ-Link Sulfo-NHS-LC-biotin (Thermo Fisher Scientific) was added and the wells were incubated for 1 h. After washing, 1.0 μg/mL HRP-conjugated streptavidin was added and incubated for 1 h. After washing, 1-step Ultra ELISA substrate (Thermo Fisher Scientific) was added to each well. The reaction was stopped by adding 2 M H_2_SO_4_, and the optical density at 450 nm was measured using a 96-well spectrophotometer (Spectra Rainbow Thermo; TECAN, Männedorf, Switzerland).

To detect OVA-specific IgG1 and IgG2a in the mouse serum, the wells of the 96-well ELISA plates were coated overnight with 5.0 μg/mL OVA (Sigma-Aldrich) in PBS, and the non-specific binding sites were blocked as described above. Diluted serum samples (1:125 dilution for IgG1 and 1:25 dilution for IgG2a) were added to the wells and incubated for 2 h. After washing with 0.05% Tween 20 in PBS, 1.0 μg/mL HRP-conjugated goat anti-mouse IgG1 (SouthernBiotech, Birmingham, AL, USA) or 1.0 μg/mL HRP-conjugated goat anti-mouse IgG2a (SouthernBiotech) was added and the wells were incubated for 1 h. After washing, 1-step Ultra ELISA substrate was added to each well, and the reaction was stopped with 2 M H_2_SO_4_. Optical density was measured at 450 nm, as described previously.

### RT-qPCR

Total RNA was extracted on day 22 from the NALTs and CLNs of allergic rhinitis-induced mice, as described above, using TRIzol reagent (Thermo Fisher Scientific). cDNA was synthesized using ReverTra Ace qPCR RT Master Mix (Toyobo, Osaka, Japan) and subjected to RT-qPCR using THUNDERBIRD SYBR qPCR Mix (Toyobo). The expression of each mRNA was normalized to that of β-actin using the ∆∆Ct method, according to the manufacturer’s instructions (CFX96 Touch Real-Time PCR Detection System; Bio-Rad Laboratories Inc., Hercules, CA, USA). The primer sets used were as follows: β-actin, 5′-CATCCGTAAAGACCTCTATGCCAAC-3′ and 5′-ATGGAGCCACCGATCCACA-3′; IL-4, 5′-TCTCGAATGTAC-CAGGAGCCATATC-3′ and 5′-AGCACCTTGGAAGCCCTACAGA-3′; IL-5, 5′-AGCACAGTGGTGAAAGAGACCTT-3′ and 5′-TCCAATGCATAGCTGGTGATTT-3′; IL-10, 5′-CGGGAAGACAATAACTGCACCC-3′ and 5′-CGGTTAGCAGTATGTTGTCCAGC-3′; and Foxp3, 5′-AGAAGTGGTGCAGTCTCTGG-3′ and 5′-AGAGCTCTTGTCCATTGAGGC-3′.

### Immunohistochemical staining of normal human tissues

The use of human tissue sections was approved by the ethics committee of the Chiba University and all research was performed in accordance with the guidelines. Informed consent was taken from the donors for this study. In brief, paraffin-embedded normal human tissue sections were deparaffinized in xylene, rehydrated in ethanol, and retrieved by boiling in 10 mM Tris–HCl buffer (pH 8.0) containing 1 mM EDTA for 20 min in a microwave oven. Endogenous peroxidase was inactivated using methanol containing 0.3% hydrogen peroxide for 30 min. After blocking non-specific binding sites with 1% BSA in PBS, the sections were incubated with 10 μg/mL SF1 in PBS containing 0.1% BSA at 4 °C overnight. After washing, the sections were incubated with HRP-conjugated goat anti-mouse IgG (Thermo Fisher Scientific) diluted 1:100 with 5% BSA in PBS for 60 min. After washing with PBS containing 0.1% BSA, the color reaction was developed in PBS containing 0.2% 3,3′-diaminobenzidine (Dojindo Laboratories, Kumamoto, Japan) and 0.02% hydrogen peroxide for 7 min. The sections were counterstained with hematoxylin. Slides were observed under a fluorescence microscope (BZ-9000; KEYENCE).

### Statistical analysis

Student’s *t-*test was used to determine the statistical significance between the experimental groups. Statistical significance was set at P < 0.05.

### Supplementary Information

Supplementary Information.

## Data Availability

All data are available in the main manuscript and the Supporting Information file.
