# Supplementary material for: A novel monoclonal antibody against 6-sulfo sialyl Lewis x glycans attenuates murine allergic rhinitis by suppressing Th2 immune responses
Source: Sci Rep. 2023 Sep 21;13:15740. doi: 10.1038/s41598-023-43017-w (PMC10514285; doi:10.1038/s41598-023-43017-w)
Supplement: Supplementary file 1 — Supplementary Information. [file 41598_2023_43017_MOESM1_ESM.pdf]

Supplementary Information

A novel monoclonal antibody against 6-sulfo sialyl Lewis x glycans attenuates murine allergic rhinitis by suppressing Th2 immune responses

Wei Liu[1], Wei Xiong[1], Wenxin Liu[1], Jotaro Hirakawa[1] and Hiroto Kawashima[1]*

[1]Laboratory of Microbiology and Immunology, Graduate School of Pharmaceutical Sciences, Chiba University, Chiba 260-8675, Japan

*Corresponding author: Hiroto Kawashima, Ph.D., Laboratory of Microbiology and Immunology, Graduate School of Pharmaceutical Sciences, Chiba University, 1-8-1 Inohana, Chuo-ku, Chiba 260-8675, Japan, Phone: +81-43-226-2926, FAX: +81-43-226-2927, Email: h-kawashima@chiba-u.jp

This file contains the following materials:

Supplementary Table S1

Supplementary Figure S1

Supplementary Table S1. Binding of SF1 to glycans displayed on a glycan array of the Consortium for Functional Glycomics

Glycan Number	Glycan Structure	Average RFU[a]	StDEV[b]	% CV[c]
1	Galα-Sp8[d]	5	4	72
2	Glcα-Sp8	13	3	25
3	Manα-Sp8	3	5	162
4	GalNAcα-Sp8	9	5	55
5	GalNAcα-Sp15	6	3	43
6	Fucα-Sp8	14	12	88
7	Fucα-Sp9	5	4	75
8	Rhaα-Sp8	5	7	159
9	Neu5Acα-Sp8	5	3	65
10	Neu5Acα-Sp11	6	2	40
11	Neu5Acβ-Sp8	22	6	27
12	Galβ-Sp8	7	5	69
13	Glcβ-Sp8	4	2	52
14	Manβ-Sp8	6	7	119
15	GalNAcβ-Sp8	11	2	19
16	GlcNAcβ-Sp0	5	3	55
17	GlcNAcβ-Sp8	4	3	74
18	GlcN(Gc)β-Sp8	5	3	47
19	Galβ1-4GlcNAcβ1-6(Galβ1-4GlcNAcβ1-3)GalNAcα-Sp8	8	10	115
20	Galβ1-4GlcNAcβ1-6(Galβ1-4GlcNAcβ1-3)GalNAc-Sp14	2	4	202
21	GlcNAcβ1-6(GlcNAcβ1-4)(GlcNAcβ1-3)GlcNAc-Sp8	5	5	106
22	6S(3S)Galβ1-4(6S)GlcNAcβ-Sp0	9	4	45
23	6S(3S)Galβ1-4GlcNAcβ-Sp0	4	4	99
24	(3S)Galβ1-4(Fucα1-3)(6S)Glc-Sp0	4	5	135
25	(3S)Galβ1-4Glcβ-Sp8	14	6	45
26	(3S)Galβ1-4(6S)Glcβ-Sp0	11	4	39
27	(3S)Galβ1-4(6S)Glcβ-Sp8	7	5	80
28	(3S)Galβ1-3(Fucα1-4)GlcNAcβ-Sp8	11	5	44
29	(3S)Galβ1-3GalNAcα-Sp8	14	14	100
30	(3S)Galβ1-3GlcNAcβ-Sp0	7	5	77
31	(3S)Galβ1-3GlcNAcβ-Sp8	13	10	80
32	(3S)Galβ1-4(Fucα1-3)GlcNAc-Sp0	4	3	74
33	(3S)Galβ1-4(Fucα1-3)GlcNAc-Sp8	4	2	53
34	(3S)Galβ1-4(6S)GlcNAcβ-Sp0	2	3	117
35	(3S)Galβ1-4(6S)GlcNAcβ-Sp8	10	8	77
36	(3S)Galβ1-4GlcNAcβ-Sp0	11	3	25
37	(3S)Galβ1-4GlcNAcβ-Sp8	5	5	100
38	(3S)Galβ-Sp8	6	6	101
39	(6S)(4S)Galβ1-4GlcNAcβ-Sp0	6	5	76
40	(4S)Galβ1-4GlcNAcβ-Sp8	11	6	54
41	(6P)Manα-Sp8	10	4	42
42	(6S)Galβ1-4Glcβ-Sp0	4	4	98

43	(6S)Galβ1-4Glcβ-Sp8	7	5	66
44	(6S)Galβ1-4GlcNAcβ-Sp8	11	12	107
45	(6S)Galβ1-4(6S)Glcβ-Sp8	5	8	152
46	Neu5Acα2-3(6S)Galβ1-4GlcNAcβ-Sp8	4	3	77
47	(6S)GlcNAcβ-Sp8	9	4	48
48	Neu5,9Ac$_2$α-Sp8	6	5	81
49	Neu5,9Ac$_2$α2-6Galβ1-4GlcNAcβ-Sp8	6	11	191
50	Manα1-6(Manα1-3)Manβ1-4GlcNAcβ1-4GlcNAcβ-Sp12	15	14	90
51	Manα1-6(Manα1-3)Manβ1-4GlcNAcβ1-4GlcNAcβ-Sp13	14	7	50
52	GlcNAcβ1-2Manα1-6(GlcNAcβ1-2Manα1-3)Manβ1-4GlcNAcβ1-4GlcNAcβ-Sp12	8	8	94
53	GlcNAcβ1-2Manα1-6(GlcNAcβ1-2Manα1-3)Manβ1-4GlcNAcβ1-4GlcNAcβ-Sp13	4	1	39
54	Galβ1-4GlcNAcβ1-2Manα1-6(Galβ1-4GlcNAcβ1-2Manα1-3)Manβ1-4GlcNAcβ1-4GlcNAcβ-Sp12	3	2	75
55	Neu5Acα2-6Galβ1-4GlcNAcβ1-2Manα1-6(Neu5Acα2-6Galβ1-4GlcNAcβ1-2Manα1-3)Manβ1-4GlcNAcβ1-4GlcNAcβ-Sp12	0	2	-385
56	Neu5Acα2-6Galβ1-4GlcNAcβ1-2Manα1-6(Neu5Acα2-6Galβ1-4GlcNAcβ1-2Manα1-3)Manβ1-4GlcNAcβ1-4GlcNAcβ-Sp13	4	6	139
57	Neu5Acα2-6Galβ1-4GlcNAcβ1-2Manα1-6(Neu5Acα2-6Galβ1-4GlcNAcβ1-2Man-α1-3)Manβ1-4GlcNAcβ1-4GlcNAcβ-Sp21	2	6	249
58	Neu5Acα2-6Galβ1-4GlcNAcβ1-2Manα1-6(Neu5Acα2-6Galβ1-4GlcNAcβ1-2Manα1-3)Manβ1-4GlcNAcβ1-4GlcNAcβ-Sp24	14	8	59
59	Fucα1-2Galβ1-3GalNAcβ1-3Galα-Sp9	10	1	6
60	Fucα1-2Galβ1-3GalNAcβ1-3Galα1-4Galβ1-4Glcβ-Sp9	10	11	102
61	Fucα1-2Galβ1-3(Fucα1-4)GlcNAcβ-Sp8	5	5	98
62	Fucα1-2Galβ1-3GalNAcα-Sp8	12	2	18
63	Fucα1-2Galβ1-3GalNAcα-Sp14	12	13	104
64	Fucα1-2Galβ1-3GalNAcβ1-4(Neu5Acα2-3)Galβ1-4Glcβ-Sp0	13	4	32
65	Fucα1-2Galβ1-3GalNAcβ1-4(Neu5Acα2-3)Galβ1-4Glcβ-Sp9	0	2	438
66	Fucα1-2Galβ1-3GlcNAcβ1-3Galβ1-4Glcβ-Sp8	11	8	76
67	Fucα1-2Galβ1-3GlcNAcβ1-3Galβ1-4Glcβ-Sp10	5	1	26
68	Fucα1-2Galβ1-3GlcNAcβ-Sp0	10	3	32
69	Fucα1-2Galβ1-3GlcNAcβ-Sp8	8	5	60
70	Fucα1-2Galβ1-4(Fucα1-3)GlcNAcβ1-3Galβ1-4(Fucα1-3)GlcNAcβ-Sp0	6	3	50
71	Fucα1-2Galβ1-4(Fucα1-3)GlcNAcβ1-3Galβ1-4(Fucα1-3)GlcNAcβ1-3Galβ1-4(Fucα1-3)GlcNAcβ-Sp0	6	5	82
72	Fucα1-2Galβ1-4(Fucα1-3)GlcNAcβ-Sp0	11	8	70
73	Fucα1-2Galβ1-4(Fucα1-3)GlcNAcβ-Sp8	2	3	142
74	Fucα1-2Galβ1-4GlcNAcβ1-3Galβ1-4GlcNAcβ-Sp0	6	5	74
75	Fucα1-2Galβ1-4GlcNAcβ1-3Galβ1-4GlcNAcβ1-3Galβ1-4GlcNAcβ-Sp0	6	4	58
76	Fucα1-2Galβ1-4GlcNAcβ-Sp0	3	1	42
77	Fucα1-2Galβ1-4GlcNAcβ-Sp8	2	4	187
78	Fucα1-2Galβ1-4Glcβ-Sp0	9	4	41
79	Fucα1-2Galβ-Sp8	5	4	80
80	Fucα1-3GlcNAcβ-Sp8	13	9	65
81	Fucα1-4GlcNAcβ-Sp8	4	4	109
82	Fucβ1-3GlcNAcβ-Sp8	5	3	58
83	GalNAcα1-3(Fucα1-2)Galβ1-3GlcNAcβ-Sp0	6	4	60
84	GalNAcα1-3(Fucα1-2)Galβ1-4(Fucα1-3)GlcNAcβ-Sp0	6	4	62
85	(3S)Galβ1-4(Fucα1-3)Glcβ-Sp0	5	1	19
86	GalNAcα1-3(Fucα1-2)Galβ1-4GlcNAcβ-Sp0	5	0	5

87	GalNAcα1-3(Fucα1-2)Galβ1-4GlcNAcβ-Sp8	4	5	123
88	GalNAcα1-3(Fucα1-2)Galβ1-4Glcβ-Sp0	5	4	78
89	GlcNAcβ1-3Galβ1-3GalNAcα-Sp8	10	3	29
90	GalNAcα1-3(Fucα1-2)Galβ-Sp8	7	5	77
91	GalNAcα1-3(Fucα1-2)Galβ-Sp18	10	8	79
92	GalNAcα1-3GalNAcβ-Sp8	7	3	49
93	GalNAcα1-3Galβ-Sp8	5	6	115
94	GalNAcα1-4(Fucα1-2)Galβ1-4GlcNAcβ-Sp8	5	3	60
95	GalNAcβ1-3GalNAcα-Sp8	3	2	68
96	GalNAcβ1-3(Fucα1-2)Galβ-Sp8	11	6	56
97	GalNAcβ1-3Galα1-4Galβ1-4GlcNAcβ-Sp0	10	5	54
98	GalNAcβ1-4(Fucα1-3)GlcNAcβ-Sp0	15	5	33
99	GalNAcβ1-4GlcNAcβ-Sp0	0	2	351
100	GalNAcβ1-4GlcNAcβ-Sp8	7	9	129
101	Galα1-2Galβ-Sp8	5	3	49
102	Galα1-3(Fucα1-2)Galβ1-3GlcNAcβ-Sp0	11	9	81
103	Galα1-3(Fucα1-2)Galβ1-3GlcNAcβ-Sp8	4	6	170
104	Galα1-3(Fucα1-2)Galβ1-4(Fucα1-3)GlcNAcβ-Sp0	10	12	116
105	Galα1-3(Fucα1-2)Galβ1-4(Fucα1-3)GlcNAcβ-Sp8	4	4	99
106	Galα1-3(Fucα1-2)Galβ1-4GlcNAc-Sp0	6	6	102
107	Galα1-3(Fucα1-2)Galβ1-4Glcβ-Sp0	13	16	122
108	Galα1-3(Fucα1-2)Galβ-Sp8	3	5	199
109	Galα1-3(Fucα1-2)Galβ-Sp18	4	2	41
110	Galα1-4(Galα1-3)Galβ1-4GlcNAcβ-Sp8	9	11	123
111	Galα1-3GalNAcα-Sp8	12	5	40
112	Galα1-3GalNAcα-Sp16	3	2	68
113	Galα1-3GalNAcβ-Sp8	15	6	44
114	Galα1-3Galβ1-4(Fucα1-3)GlcNAcβ-Sp8	5	3	64
115	Galα1-3Galβ1-3GlcNAcβ-Sp0	9	11	115
116	Galα1-3Galβ1-4GlcNAcβ-Sp8	7	2	32
117	Galα1-3Galβ1-4Glcβ-Sp0	9	8	91
118	Galα1-3Galβ1-4Glc-Sp10	20	7	35
119	Galα1-3Galβ-Sp8	1	2	192
120	Galα1-4(Fucα1-2)Galβ1-4GlcNAcβ-Sp8	10	10	102
121	Galα1-4Galβ1-4GlcNAcβ-Sp0	5	3	62
122	Galα1-4Galβ1-4GlcNAcβ-Sp8	9	8	82
123	Galα1-4Galβ1-4Glcβ-Sp0	8	7	89
124	Galα1-4GlcNAcβ-Sp8	15	6	40
125	Galα1-6Glcβ-Sp8	4	7	152
126	Galβ1-2Galβ-Sp8	5	2	40
127	Galβ1-3(Fucα1-4)GlcNAcβ1-3Galβ1-4(Fucα1-3)GlcNAcβ-Sp0	4	3	72
128	Galβ1-3GlcNAcβ1-3Galβ1-4(Fucα1-3)GlcNAcβ-Sp0	2	4	161
129	Galβ1-3(Fucα1-4)GlcNAc-Sp0	5	2	48
130	Galβ1-3(Fucα1-4)GlcNAc-Sp8	8	7	87
131	Fucα1-4(Galβ1-3)GlcNAcβ-Sp8	10	3	29
132	Galβ1-4GlcNAcβ1-6GalNAcα-Sp8	11	9	82
133	Galβ1-4GlcNAcβ1-6GalNAc-Sp14	9	6	71

134	GlcNAcβ1-6(Galβ1-3)GalNAcα-Sp8	6	4	57
135	GlcNAcβ1-6(Galβ1-3)GalNAcα-Sp14	9	6	75
136	Neu5Acα2-6(Galβ1-3)GalNAcα-Sp8	6	3	54
137	Neu5Acα2-6(Galβ1-3)GalNAcα-Sp14	6	3	53
138	Neu5Acβ2-6(Galβ1-3)GalNAcα-Sp8	15	10	64
139	Neu5Acα2-6(Galβ1-3)GlcNAcβ1-4Galβ1-4Glcβ-Sp10	6	2	30
140	Galβ1-3GalNAcα-Sp8	11	5	48
141	Galβ1-3GalNAcα-Sp14	6	1	19
142	Galβ1-3GalNAcα-Sp16	3	2	61
143	Galβ1-3GalNAcβ-Sp8	8	3	38
144	Galβ1-3GalNAcβ1-3Galα1-4Galβ1-4Glcβ-Sp0	5	5	98
145	Galβ1-3GalNAcβ1-4(Neu5Acα2-3)Galβ1-4Glcβ-Sp0	5	5	93
146	Galβ1-3GalNAcβ1-4Galβ1-4Glcβ-Sp8	6	9	153
147	Galβ1-3Galβ-Sp8	2	4	265
148	Galβ1-3GlcNAcβ1-3Galβ1-4GlcNAcβ-Sp0	9	7	76
149	Galβ1-3GlcNAcβ1-3Galβ1-4Glcβ-Sp10	6	3	58
150	Galβ1-3GlcNAcβ-Sp0	3	2	62
151	Galβ1-3GlcNAcβ-Sp8	10	3	27
152	Galβ1-4(Fucα1-3)GlcNAcβ-Sp0	11	7	63
153	Galβ1-4(Fucα1-3)GlcNAcβ-Sp8	6	2	33
154	Galβ1-4(Fucα1-3)GlcNAcβ1-3Galβ1-4(Fucα1-3)GlcNAcβ-Sp0	1	2	164
155	Galβ1-4(Fucα1-3)GlcNAcβ1-3Galβ1-4(Fucα1-3)GlcNAcβ1-3Galβ1-4(Fucα1-3)GlcNAcβ-Sp0	8	5	57
156	Galβ1-4(6S)Glcβ-Sp0	4	1	37
157	Galβ1-4(6S)Glcβ-Sp8	10	4	37
158	Galβ1-4GalNAcα1-3(Fucα1-2)Galβ1-4GlcNAcβ-Sp8	13	6	43
159	Galβ1-4GalNAcβ1-3(Fucα1-2)Galβ1-4GlcNAcβ-Sp8	5	2	42
160	Galβ1-4GlcNAcβ1-3GalNAcα-Sp8	15	11	72
161	Galβ1-4GlcNAcβ1-3GalNAc-Sp14	8	5	67
162	Galβ1-4GlcNAcβ1-3Galβ1-4(Fucα1-3)GlcNAcβ1-3Galβ1-4(Fucα1-3)GlcNAcβ-Sp0	10	2	21
163	Galβ1-4GlcNAcβ1-3Galβ1-4GlcNAcβ1-3Galβ1-4GlcNAcβ-Sp0	8	3	41
164	Galβ1-4GlcNAcβ1-3Galβ1-4GlcNAcβ-Sp0	15	8	52
165	Galβ1-4GlcNAcβ1-3Galβ1-4Glcβ-Sp0	3	4	144
166	Galβ1-4GlcNAcβ1-3Galβ1-4Glcβ-Sp8	6	6	105
167	Galβ1-4GlcNAcβ1-6(Galβ1-3)GalNAcα-Sp8	13	9	66
168	Galβ1-4GlcNAcβ1-6(Galβ1-3)GalNAc-Sp14	1	2	411
169	Galβ1-4GlcNAcβ-Sp0	19	11	60
170	Galβ1-4GlcNAcβ-Sp8	5	3	47
171	Galβ1-4GlcNAcβ-Sp23	6	5	89
172	Galβ1-4Glcβ-Sp0	10	2	24
173	Galβ1-4Glcβ-Sp8	5	3	62
174	GlcNAcα1-3Galβ1-4GlcNAcβ-Sp8	7	3	49
175	GlcNAcα1-6Galβ1-4GlcNAcβ-Sp8	12	5	44
176	GlcNAcβ1-2Galβ1-3GalNAcα-Sp8	8	7	86
177	GlcNAcβ1-6(GlcNAcβ1-3)GalNAcα-Sp8	4	4	94
178	GlcNAcβ1-6(GlcNAcβ1-3)GalNAcα-Sp14	8	8	105
179	GlcNAcβ1-6(GlcNAcβ1-3)Galβ1-4GlcNAcβ-Sp8	5	3	60

180	GlcNAcβ1-3GalNAcα-Sp8	15	7	47
181	GlcNAcβ1-3GalNAcα-Sp14	6	4	67
182	GlcNAcβ1-3Galβ-Sp8	6	5	83
183	GlcNAcβ1-3Galβ1-4GlcNAcβ-Sp0	9	8	93
184	GlcNAcβ1-3Galβ1-4GlcNAcβ-Sp8	13	8	59
185	GlcNAcβ1-3Galβ1-4GlcNAcβ1-3Galβ1-4GlcNAcβ-Sp0	3	3	104
186	GlcNAcβ1-3Galβ1-4Glcβ-Sp0	2	1	39
187	GlcNAcβ1-4-MDPLys	8	3	38
188	GlcNAcβ1-6(GlcNAcβ1-4)GalNAcα-Sp8	7	7	96
189	GlcNAcβ1-4Galβ1-4GlcNAcβ-Sp8	6	6	107
190	GlcNAcβ1-4GlcNAcβ1-4GlcNAcβ1-4GlcNAcβ1-4GlcNAcβ1-4GlcNAcβ1-Sp8	1	6	436
191	GlcNAcβ1-4GlcNAcβ1-4GlcNAcβ1-4GlcNAcβ1-4GlcNAcβ1-Sp8	5	1	32
192	GlcNAcβ1-4GlcNAcβ1-4GlcNAcβ-Sp8	4	4	94
193	GlcNAcβ1-6GalNAcα-Sp8	12	9	71
194	GlcNAcβ1-6GalNAcα-Sp14	5	7	134
195	GlcNAcβ1-6Galβ1-4GlcNAcβ-Sp8	7	5	78
196	Glcα1-4Glcβ-Sp8	9	4	40
197	Glcα1-4Glcα-Sp8	11	9	84
198	Glcα1-6Glcα1-6Glcβ-Sp8	2	2	89
199	Glcβ1-4Glcβ-Sp8	5	4	76
200	Glcβ1-6Glcβ-Sp8	7	5	75
201	G-ol-Sp8	5	5	94
202	GlcAα-Sp8	6	6	101
203	GlcAβ-Sp8	5	2	41
204	GlcAβ1-3Galβ-Sp8	8	1	16
205	GlcAβ1-6Galβ-Sp8	10	6	68
206	KDNα2-3Galβ1-3GlcNAcβ-Sp0	6	6	98
207	KDNα2-3Galβ1-4GlcNAcβ-Sp0	11	5	45
208	Manα1-2Manα1-2Manα1-3Manα-Sp9	4	6	139
209	Manα1-2Manα1-6(Manα1-2Manα1-3)Manα-Sp9	6	2	39
210	Manα1-2Manα1-3Manα-Sp9	6	4	72
211	Manα1-6(Manα1-2Manα1-3)Manα1-6(Manα1-2Manα1-3)Manβ1-4GlcNAcβ1-4GlcNAcβ-Sp12	7	7	91
212	Manα1-2Manα1-6(Manα1-3)Manα1-6(Manα1-2Manα1-2Manα1-3)Manβ1-4GlcNAcβ1-4GlcNAcβ-Sp12	15	6	43
213	Manα1-2Manα1-6(Manα1-2Manα1-3)Manα1-6(Manα1-2Manα1-2Manα1-3)Manβ1-4GlcNAcβ1-4GlcNAcβ-Sp12	19	17	93
214	Manα1-6(Manα1-3)Manα-Sp9	0	4	-3791
215	Manα1-2Manα1-2Manα1-6(Manα1-3)Manα-Sp9	5	7	154
216	Manα1-6(Manα1-3)Manα1-6(Manα1-2Manα1-3)Manβ1-4GlcNAcβ1-4GlcNAcβ-Sp12	12	2	21
217	Manα1-6(Manα1-3)Manα1-6(Manα1-3)Manβ1-4GlcNAcβ1-4GlcNAcβ-Sp12	6	6	109
218	Manβ1-4GlcNAcβ-Sp0	5	1	17
219	Neu5Acα2-3Galβ1-4GlcNAcβ1-3Galβ1-4(Fucα1-3)GlcNAcβ-Sp0	4	7	169
220	(3S)Galβ1-4(Fucα1-3)(6S)GlcNAcβ-Sp8	3	4	128
221	Fucα1-2(6S)Galβ1-4GlcNAcβ-Sp0	0	4	-4657
222	Fucα1-2Galβ1-4(6S)GlcNAcβ-Sp8	5	1	28
223	Fucα1-2(6S)Galβ1-4(6S)Glcβ-Sp0	13	7	52
224	Neu5Acα2-3Galβ1-3GalNAcα-Sp8	8	6	77

225	Neu5Acα2-3Galβ1-3GalNAcα-Sp14	5	6	119
226	GalNAcβ1-4(Neu5Acα2-8Neu5Acα2-8Neu5Acα2-8Neu5Acα2-3)Galβ1-4Glcβ-Sp0	4	3	78
227	GalNAcβ1-4(Neu5Acα2-8Neu5Acα2-8Neu5Acα2-3)Galβ1-4Glcβ-Sp0	9	6	62
228	Neu5Acα2-8Neu5Acα2-8Neu5Acα2-3Galβ1-4Glcβ-Sp0	12	7	54
229	GalNAcβ1-4(Neu5Acα2-8Neu5Acα2-3)Galβ1-4Glcβ–Sp0	12	1	12
230	Neu5Acα2-8Neu5Acα2-8Neu5Acα-Sp8	1	0	61
231	Neu5Acα2-3(6S)Galβ1-4(Fucα1-3)GlcNAcβ-Sp8	10	10	99
232	GalNAcβ1-4(Neu5Acα2-3)Galβ1-4GlcNAcβ-Sp0	3	2	59
233	GalNAcβ1-4(Neu5Acα2-3)Galβ1-4GlcNAcβ-Sp8	0	3	-1730
234	GalNAcβ1-4(Neu5Acα2-3)Galβ1-4Glcβ-Sp0	5	4	77
235	Neu5Acα2-3Galβ1-3GalNAcβ1-4(Neu5Acα2-3)Galβ1-4Glcβ-Sp0	12	5	36
236	Neu5Acα2-6(Neu5Acα2-3)GalNAcα-Sp8	15	12	77
237	Neu5Acα2-3GalNAcα-Sp8	5	2	37
238	Neu5Acα2-3GalNAcβ1-4GlcNAcβ-Sp0	6	6	102
239	Neu5Acα2-3Galβ1-3(6S)GlcNAc-Sp8	10	9	92
240	Neu5Acα2-3Galβ1-3(Fucα1-4)GlcNAcβ-Sp8	12	6	48
241	Neu5Acα2-3Galβ1-3(Fucα1-4)GlcNAcβ1-3Galβ1-4(Fucα1-3)GlcNAcβ-Sp0	6	4	68
242	Neu5Acα2-3Galβ1-4(Neu5Acα2-3Galβ1-3)GlcNAcβ-Sp8	7	8	118
243	Neu5Acα2-3Galβ1-3(6S)GalNAcα-Sp8	5	7	151
244	Neu5Acα2-6(Neu5Acα2-3Galβ1-3)GalNAcα-Sp8	8	6	71
245	Neu5Acα2-6(Neu5Acα2-3Galβ1-3)GalNAcα-Sp14	4	2	46
246	Neu5Acα2-3Galβ-Sp8	6	7	116
247	Neu5Acα2-3Galβ1-3GalNAcβ1-3Galα1-4Galβ1-4Glcβ-Sp0	6	5	81
248	Neu5Acα2-3Galβ1-3GlcNAcβ1-3Galβ1-4GlcNAcβ-Sp0	6	7	121
249	Fucα1-2(6S)Galβ1-4Glcβ-Sp0	5	9	178
250	Neu5Acα2-3Galβ1-3GlcNAcβ-Sp0	11	8	77
251	Neu5Acα2-3Galβ1-3GlcNAcβ-Sp8	-1	2	-214
252	Neu5Acα2-3Galβ1-4(6S)GlcNAcβ-Sp8	24	6	25
253	Neu5Acα2-3Galβ1-4(Fucα1-3)(6S)GlcNAcβ-Sp8	3920	203	5
254	Neu5Acα2-3Galβ1-4(Fucα1-3)GlcNAcβ1-3Galβ1-4(Fucα1-3)GlcNAcβ1-3Galβ1-4(Fucα1-3)GlcNAcβ-Sp0	17	6	38
255	Neu5Acα2-3Galβ1-4(Fucα1-3)GlcNAcβ-Sp0	7	4	50
256	Neu5Acα2-3Galβ1-4(Fucα1-3)GlcNAcβ-Sp8	12	4	36
257	Neu5Acα2-3Galβ1-4(Fucα1-3)GlcNAcβ1-3Galβ-Sp8	10	9	91
258	Neu5Acα2-3Galβ1-4(Fucα1-3)GlcNAcβ1-3Galβ1-4GlcNAcβ-Sp8	18	12	66
259	Neu5Acα2-3Galβ1-4GlcNAcβ1-3Galβ1-4GlcNAcβ1-3Galβ1-4GlcNAcβ-Sp0	2	2	94
260	Neu5Acα2-3Galβ1-4GlcNAcβ-Sp0	6	4	65
261	Neu5Acα2-3Galβ1-4GlcNAcβ-Sp8	10	10	92
262	Neu5Acα2-3Galβ1-4GlcNAcβ1-3Galβ1-4GlcNAcβ-Sp0	3	4	108
263	Fucα1-2Galβ1-4(6S)Glcβ-Sp0	2	2	99
264	Neu5Acα2-3Galβ1-4Glcβ-Sp0	9	4	51
265	Neu5Acα2-3Galβ1-4Glcβ-Sp8	9	6	66
266	Neu5Acα2-6GalNAcα-Sp8	5	2	42
267	Neu5Acα2-6GalNAcβ1-4GlcNAcβ-Sp0	9	8	82
268	Neu5Acα2-6Galβ1-4(6S)GlcNAcβ-Sp8	7	3	46
269	Neu5Acα2-6Galβ1-4GlcNAcβ-Sp0	8	4	46
270	Neu5Acα2-6Galβ1-4GlcNAcβ-Sp8	11	9	86

271	Neu5Acα2-6Galβ1-4GlcNAcβ1-3Galβ1-4(Fucα1-3)GlcNAcβ1-3Galβ1-4(Fucα1-3)GlcNAcβ-Sp0	3	2	58
272	Neu5Acα2-6Galβ1-4GlcNAcβ1-3Galβ1-4GlcNAcβ-Sp0	18	7	41
273	Neu5Acα2-6Galβ1-4Glcβ-Sp0	6	2	36
274	Neu5Acα2-6Galβ1-4Glcβ-Sp8	5	2	39
275	Neu5Acα2-6Galβ-Sp8	7	4	54
276	Neu5Acα2-8Neu5Acα-Sp8	9	6	60
277	Neu5Acα2-8Neu5Acα2-3Galβ1-4Glcβ-Sp0	9	5	55
278	Galβ1-3(Fucα1-4)GlcNAcβ1-3Galβ1-3(Fucα1-4)GlcNAcβ-Sp0	1	2	157
279	Neu5Acβ2-6GalNAcα-Sp8	4	2	54
280	Neu5Acβ2-6Galβ1-4GlcNAcβ-Sp8	3	3	94
281	Neu5Gcα2-3Galβ1-3(Fucα1-4)GlcNAcβ-Sp0	9	3	37
282	Neu5Gcα2-3Galβ1-3GlcNAcβ-Sp0	9	4	42
283	Neu5Gcα2-3Galβ1-4(Fucα1-3)GlcNAcβ-Sp0	15	11	73
284	Neu5Gcα2-3Galβ1-4GlcNAcβ-Sp0	5	8	147
285	Neu5Gcα2-3Galβ1-4Glcβ-Sp0	9	8	94
286	Neu5Gcα2-6GalNAcα-Sp0	8	10	126
287	Neu5Gcα2-6Galβ1-4GlcNAcβ-Sp0	2	2	93
288	Neu5Gcα-Sp8	14	7	47
289	Neu5Acα2-3Galβ1-4GlcNAcβ1-6(Galβ1-3)GalNAcα-Sp14	10	5	50
290	Galβ1-3GlcNAcβ1-3Galβ1-3GlcNAcβ-Sp0	11	13	120
291	Galβ1-4(Fucα1-3)(6S)GlcNAcβ-Sp0	3	1	47
292	Galβ1-4(Fucα1-3)(6S)Glcβ-Sp0	5	4	75
293	Galβ1-4(Fucα1-3)GlcNAcβ1-3Galβ1-3(Fucα1-4)GlcNAcβ-Sp0	9	7	79
294	Galβ1-4GlcNAcβ1-3Galβ1-3GlcNAcβ-Sp0	7	3	38
295	Neu5Acα2-3Galβ1-3GlcNAcβ1-3Galβ1-3GlcNAcβ-Sp0	3	3	101
296	Neu5Acα2-3Galβ1-4GlcNAcβ1-3Galβ1-3GlcNAcβ-Sp0	5	4	80
297	4S(3S)Galβ1-4GlcNAcβ-Sp0	4	7	183
298	(6S)Galβ1-4(6S)GlcNAcβ-Sp0	5	2	44
299	(6P)Glcβ-Sp10	7	10	130
300	Neu5Acα2-3Galβ1-4(Fucα1-3)GlcNAcβ1-6(Galβ1-3)GalNAcα-Sp14	9	6	70
301	Galβ1-3Galβ1-4GlcNAcβ-Sp8	6	1	10
302	Neu5Acα2-6Galβ1-4GlcNAcβ1-2Manα1-6(Galβ1-4GlcNAcβ1-2Manα1-3)Manβ1-4GlcNAcβ1-4GlcNAcβ-Sp12	6	3	49
303	Galβ1-4GlcNAcβ1-6(Galβ1-4GlcNAcβ1-3)Galβ1-4GlcNAc-Sp0	14	8	54
304	GlcNAcβ1-6(Galβ1-4GlcNAcβ1-3)Galβ1-4GlcNAc-Sp0	7	2	34
305	Galβ1-4GlcNAcα1-6Galβ1-4GlcNAcβ-Sp0	3	1	40
306	Galβ1-4GlcNAcβ1-6Galβ1-4GlcNAcβ-Sp0	10	5	47
307	GalNAcβ1-3Galβ-Sp8	10	10	92
308	GlcAβ1-3GlcNAcβ-Sp8	9	8	89
309	Neu5Acα2-6Galβ1-4GlcNAcβ1-2Manα1-6(GlcNAcβ1-2Manα1-3)Manβ1-4GlcNAcβ1-4GlcNAcβ-Sp12	9	5	50
310	GlcNAcβ1-3Man-Sp10	9	5	55
311	GlcNAcβ1-4GlcNAcβ-Sp10	7	3	40
312	GlcNAcβ1-4GlcNAcβ-Sp12	5	5	95
313	MurNAcβ1-4GlcNAcβ-Sp10	12	4	37
314	Manα1-6Manβ-Sp10	7	2	27
315	Manα1-6(Manα1-3)Manα1-6(Manα1-3)Manβ-Sp10	18	10	59

316	Manα1-2Manα1-6(Manα1-3)Manα1-6(Manα1-2Manα1-2Manα1-3)Manα-Sp9	9	3	38
317	Manα1-2Manα1-6(Manα1-2Manα1-3)Manα1-6(Manα1-2Manα1-2Manα1-3)Manα-Sp9	6	5	78
318	Neu5Acα2-3Galβ1-4GlcNAcβ1-6(Neu5Acα2-3Galβ1-3)GalNAcα-Sp14	4	3	78
319	Neu5Acα2-6Galβ1-4GlcNAcβ1-2Manα1-6(Neu5Acα2-3Galβ1-4GlcNAcβ1-2Manα1-3)Manβ1-4GlcNAcβ1-4GlcNAcβ-Sp12	3	2	59
320	Galβ1-4GlcNAcβ1-2Manα1-6(Neu5Acα2-6Galβ1-4GlcNAcβ1-2Manα1-3)Manβ1-4GlcNAcβ1-4GlcNAcβ-Sp12	6	4	69
321	GlcNAcβ1-2Manα1-6(Neu5Acα2-6Galβ1-4GlcNAcβ1-2Manα1-3)Manβ1-4GlcNAcβ1-4GlcNAcβ-Sp12	5	4	92
322	Neu5Acα2-8Neu5Acβ-Sp17	3	3	93
323	Neu5Acα2-8Neu5Acα2-8Neu5Acβ-Sp8	9	11	113
324	Neu5Gcβ2-6Galβ1-4GlcNAc-Sp8	8	8	98
325	Galβ1-3GlcNAcβ1-2Manα1-6(Galβ1-3GlcNAcβ1-2Manα1-3)Manβ1-4GlcNAcβ1-4GlcNAcβ-Sp19	7	9	125
326	Neu5Acα2-3Galβ1-4GlcNAcβ1-2Manα1-6(Neu5Acα2-3Galβ1-4GlcNAcβ1-2Manα1-3)Manβ1-4GlcNAcβ1-4GlcNAcβ-Sp12	4	3	69
327	Neu5Acα2-3Galβ1-4GlcNAcβ1-2Manα1-6(Neu5Acα2-6Galβ1-4GlcNAcβ1-2Manα1-3)Manβ1-4GlcNAcβ1-4GlcNAcβ-Sp12	7	10	144
328	Galβ1-4(Fucα1-3)GlcNAcβ1-2Manα1-6(Galβ1-4(Fucα1-3)GlcNAcβ1-2Manα1-3)Manβ1-4GlcNAcβ1-4GlcNAcβ-Sp20	6	8	117
329	Neu5,9Ac$_2$α2-3Galβ1-4GlcNAcβ-Sp0	9	6	72
330	Neu5,9Ac$_2$α2-3Galβ1-3GlcNAcβ-Sp0	9	7	74
331	Neu5Acα2-6Galβ1-4GlcNAcβ1-3Galβ1-3GlcNAcβ-Sp0	17	7	42
332	Neu5Acα2-3Galβ1-3(Fucα1-4)GlcNAcβ1-3Galβ1-3(Fucα1-4)GlcNAcβ-Sp0	19	13	67
333	Neu5Acα2-6Galβ1-4GlcNAcβ1-3Galβ1-4GlcNAcβ1-3Galβ1-4GlcNAcβ-Sp0	2	3	181
334	Galα1-4Galβ1-4GlcNAcβ1-3Galβ1-4Glcβ-Sp0	2	2	109
335	GalNAcβ1-3Galα1-4Galβ1-4GlcNAcβ1-3Galβ1-4Glcβ-Sp0	1	2	169
336	GalNAcα1-3(Fucα1-2)Galβ1-4GlcNAcβ1-3Galβ1-4GlcNAcβ-Sp0	7	6	87
337	GalNAcα1-3(Fucα1-2)Galβ1-4GlcNAcβ1-3Galβ1-4GlcNAcβ1-3Galβ1-4GlcNAcβ-Sp0	8	14	180
338	Neu5Acα2-3Galβ1-4(Fucα1-3)GlcNAcβ1-6(Neu5Acα2-3Galβ1-3)GalNAc-Sp14	7	4	55
339	GlcNAcα1-4Galβ1-4GlcNAcβ1-3Galβ1-4GlcNAcβ1-3Galβ1-4GlcNAcβ-Sp0	16	12	75
340	GlcNAcα1-4Galβ1-4GlcNAcβ-Sp0	8	7	84
341	GlcNAcα1-4Galβ1-3GlcNAcβ-Sp0	8	1	7
342	GlcNAcα1-4Galβ1-4GlcNAcβ1-3Galβ1-4Glcβ-Sp0	5	2	34
343	GlcNAcα1-4Galβ1-4GlcNAcβ1-3Galβ1-4(Fucα1-3)GlcNAcβ1-3Galβ1-4(Fucα1-3)GlcNAcβ-Sp0	3	3	115
344	GlcNAcα1-4Galβ1-4GlcNAcβ1-3Galβ1-4GlcNAcβ-Sp0	3	4	126
345	GlcNAcα1-4Galβ1-3GalNAc-Sp14	8	8	101
346	Neu5Acα2-6Galβ1-4GlcNAcβ1-2Manα1-6(Manα1-3)Manβ1-4GlcNAcβ1-4GlcNAc-Sp12	4	4	94
347	Manα1-6(Neu5Acα2-6Galβ1-4GlcNAcβ1-2Manα1-3)Manβ1-4GlcNAcβ1-4GlcNAc-Sp12	3	4	131
348	Neu5Acα2-6Galβ1-4GlcNAcβ1-2Manα1-6Manβ1-4GlcNAcβ1-4GlcNAc-Sp12	11	6	54
349	Neu5Acα2-6Galβ1-4GlcNAcβ1-2Manα1-3Manβ1-4GlcNAcβ1-4GlcNAc-Sp12	12	5	40
350	Galβ1-4GlcNAcβ1-2Manα1-3Manβ1-4GlcNAcβ1-4GlcNAc-Sp12	3	2	70
351	Galβ1-4GlcNAcβ1-2Manα1-6Manβ1-4GlcNAcβ1-4GlcNAc-Sp12	8	5	67
352	Manα1-6(Galβ1-4GlcNAcβ1-2Manα1-3)Manβ1-4GlcNAcβ1-4GlcNAcβ-Sp12	-1	3	-357
353	GlcNAcβ1-2Manα1-6(GlcNAcβ1-2Manα1-3)Manβ1-4GlcNAcβ1-4(Fucα1-6)GlcNAcβ-Sp22	3	4	139
354	Galβ1-4GlcNAcβ1-2Manα1-6(Galβ1-4GlcNAcβ1-2Manα1-3)Manβ1-4GlcNAcβ1-4(Fucα1-6)GlcNAcβ-Sp22	35	33	94
355	Galβ1-3GlcNAcβ1-2Manα1-6(Galβ1-3GlcNAcβ1-2Manα1-3)Manβ1-4GlcNAcβ1-4(Fucα1-6)GlcNAcβ-Sp22	8	5	60
356	(6S)GlcNAcβ1-3Galβ1-4GlcNAcβ-Sp0	12	8	65

357	KDNα2-3Galβ1-4(Fucα1-3)GlcNAc-Sp0	11	4	33
358	KDNα2-6Galβ1-4GlcNAc-Sp0	7	3	45
359	KDNα2-3Galβ1-4Glc-Sp0	6	4	63
360	KDNα2-3Galβ1-3GalNAcα-Sp14	4	6	157
361	Fucα1-2Galβ1-3GlcNAcβ1-2Manα1-6(Fucα1-2Galβ1-3GlcNAcβ1-2Manα1-3)Manβ1-4GlcNAcβ1-4GlcNAcβ-Sp20	9	7	76
362	Fucα1-2Galβ1-4GlcNAcβ1-2Manα1-6(Fucα1-2Galβ1-4GlcNAcβ1-2Manα1-3)Manβ1-4GlcNAcβ1-4GlcNAcβ-Sp20	10	5	52
363	Fucα1-2Galβ1-4(Fucα1-3)GlcNAcβ1-2Manα1-6(Fucα1-2Galβ1-4(Fucα1-3)GlcNAcβ1-2Manα1-3)Manβ1-4GlcNAcβ1-4GlcNAβ-Sp20	11	6	52
364	Galα1-3Galβ1-4GlcNAcβ1-2Manα1-6(Galα1-3Galβ1-4GlcNAcβ1-2Manα1-3)Manβ1-4GlcNAcβ1-4GlcNAcβ-Sp20	4	11	288
365	Galβ1-4GlcNAcβ1-2Manα1-6(Manα1-3)Manβ1-4GlcNAcβ1-4GlcNAcβ-Sp12	6	3	44
366	Fucα1-4(Galβ1-3)GlcNAcβ1-2Manα1-6(Fucα1-4(Galβ1-3)GlcNAcβ1-2Manα1-3)Manβ1-4GlcNAcβ1-4(Fucα1-6)GlcNAcβ-Sp22	10	7	67
367	Neu5Acα2-6GlcNAcβ1-4GlcNAc-Sp21	10	3	32
368	Neu5Acα2-6GlcNAcβ1-4GlcNAcβ1-4GlcNAc-Sp21	1	3	302
369	Galβ1-4(Fucα1-3)GlcNAcβ1-6(Fucα1-2Galβ1-4GlcNAcβ1-3)Galβ1-4Glc-Sp21	5	4	80
370	Galβ1-4GlcNAcβ1-2Manα1-6(Galβ1-4GlcNAcβ1-4(Galβ1-4GlcNAcβ1-2)Manα1-3)Manβ1-4GlcNAcβ1-4GlcNAc-Sp21	7	5	70
371	GalNAcα1-3(Fucα1-2)Galβ1-4GlcNAcβ1-2Manα1-6(GalNAcα1-3(Fucα1-2)Galβ1-4GlcNAcβ1-2Manα1-3)Manβ1-4GlcNAcβ1-4GlcNAcβ-Sp20	7	4	50
372	Galα1-3(Fucα1-2)Galβ1-4GlcNAcβ1-2Manα1-6(Galα1-3(Fucα1-2)Galβ1-4GlcNAcβ1-2Manα1-3)Manβ1-4GlcNAcβ1-4GlcNAcβ-Sp20	12	5	42
373	Galα1-3Galβ1-4(Fucα1-3)GlcNAcβ1-2Manα1-6(Galα1-3Galβ1-4(Fucα1-3)GlcNAcβ1-2Manα1-3)Manβ1-4GlcNAcβ1-4GlcNAcβ-Sp20	13	7	55
374	GalNAcα1-3(Fucα1-2)Galβ1-3GlcNAcβ1-2Manα1-6(GalNAcα1-3(Fucα1-2)Galβ1-3GlcNAcβ1-2Manα1-3)Manβ1-4GlcNAcβ1-4GlcNAcβ-Sp20	3	1	43
375	Galα1-3(Fuca1-2)Galβ1-3GlcNAcβ1-2Manα1-6(Galα1-3(Fucα1-2)Galβ1-3GlcNAcβ1-2Manα1-3)Manβ1-4GlcNAcβ1-4GlcNAcβ-Sp20	11	8	70
376	Fucα1-4(Fucα1-2Galβ1-3)GlcNAcβ1-2Manα1-3(Fucα1-4(Fucα1-2Galβ1-3)GlcNAcβ1-2Manα1-3)Manβ1-4GlcNAcβ1-4GlcNAcβ-Sp19	4	5	124
377	Neu5Acα2-3Galβ1-4GlcNAcβ1-3GalNAc-Sp14	2	4	153
378	Neu5Acα2-6Galβ1-4GlcNAcβ1-3GalNAc-Sp14	20	10	52
379	Neu5Acα2-3Galβ1-4(Fucα1-3)GlcNAcβ1-3GalNAcα-Sp14	14	9	66
380	GalNAcβ1-4GlcNAcβ1-2Manα1-6(GalNAcβ1-4GlcNAcβ1-2Manα1-3)Manβ1-4GlcNAcβ1-4GlcNAc-Sp12	5	5	104
381	Galβ1-3GalNAcα1-3(Fucα1-2)Galβ1-4Glc-Sp0	2	3	130
382	Galβ1-3GalNAcα1-3(Fucα1-2)Galβ1-4GlcNAc-Sp0	10	12	120
383	Galβ1-3GlcNAcβ1-3Galβ1-4GlcNAcβ1-6(Galβ1-3GlcNAcβ1-3)Galβ1-4Glcβ-Sp0	8	7	83
384	Galβ1-4(Fucα1-3)GlcNAcβ1-6(Galβ1-3GlcNAcβ1-3)Galβ1-4Glc-Sp21	10	5	53
385	Galβ1-4GlcNAcβ1-6(Fucα1-4(Fucα1-2Galβ1-3)GlcNAcβ1-3)Galβ1-4Glc-Sp21	-1	3	-357
386	Galβ1-4(Fucα1-3)GlcNAcβ1-6(Fucα1-4(Fucα1-2Galβ1-3)GlcNAcβ1-3)Galβ1-4Glc-Sp21	0	2	334
387	Galβ1-3GlcNAcβ1-3Galβ1-4(Fucα1-3)GlcNAcβ1-6(Galβ1-3GlcNAcβ1-3)Galβ1-4Glc-Sp21	7	8	113
388	Galβ1-4GlcNAcβ1-6(Galβ1-4GlcNAcβ1-2)Manα1-6(Galβ1-4GlcNAcβ1-4(Galβ1-4GlcNAcβ1-2)Manα1-3)Manβ1-4GlcNAcβ1-4GlcNAcβ-Sp21	7	5	72
389	GlcNAcβ1-2Manα1-6(GlcNAcβ1-4(GlcNAcβ1-2)Manα1-3)Manβ1-4GlcNAcβ1-4GlcNAc-Sp21	11	7	64
390	Fucα1-2Galβ1-3GalNAcα1-3(Fucα1-2)Galβ1-4Glcβ-Sp0	0	3	2674
391	Fucα1-2Galβ1-3GalNAcα1-3(Fucα1-2)Galβ1-4GlcNAcβ-Sp0	4	2	63
392	Galβ1-3GlcNAcβ1-3GalNAcα-Sp14	4	3	77
393	GalNAcβ1-4(Neu5Acα2-3)Galβ1-4GlcNAcβ1-3GalNAcα-Sp14	7	8	105
394	GalNAcα1-3(Fucα1-2)Galβ1-3GalNAcα1-3(Fucα1-2)Galβ1-4GlcNAcβ-Sp0	3	4	129
395	Galα1-3Galβ1-3GlcNAcβ1-2Manα1-6(Galα1-3Galβ1-3GlcNAcβ1-2Manα1-3)Manβ1-4GlcNAcβ1-4GlcNAc-Sp19	30	26	85

396	Galα1-3Galβ1-3(Fucα1-4)GlcNAcβ1-2Manα1-6(Galα1-3Galβ1-3(Fucα1-4)GlcNAcβ1-2Manα1-3)Manβ1-4GlcNAcβ1-4GlcNAc-Sp19	7	1	18
397	Neu5Acα2-3Galβ1-3GlcNAcβ1-2Manα1-6(Neu5Acα2-3Galβ1-3GlcNAcβ1-2Manα1-3)Manβ1-4GlcNAcβ1-4GlcNAc-Sp19	4	6	129
398	GlcNAcβ1-2Manα1-6(Galβ1-4GlcNAcβ1-2Manα1-3)Manβ1-4GlcNAcβ1-4GlcNAc-Sp12	4	2	54
399	Galβ1-4GlcNAcβ1-2Manα1-6(GlcNAcβ1-2Manα1-3)Manβ1-4GlcNAcβ1-4GlcNAc-Sp12	8	4	46
400	Neu5Acα2-3Galβ1-3GlcNAcβ1-3GalNAcα-Sp14	7	7	89
401	Fucα1-2Galβ1-4GlcNAcβ1-3GalNAcα-Sp14	8	4	53
402	Galβ1-4(Fucα1-3)GlcNAcβ1-3GalNAcα-Sp14	5	8	152
403	GalNAcα1-3GalNAcβ1-3Galα1-4Galβ1-4GlcNAcβ-Sp0	5	2	29
404	Galα1-4Galβ1-3GlcNAcβ1-2Manα1-6(Galα1-4Galβ1-3GlcNAcβ1-2Manα1-3)Manβ1-4GlcNAcβ1-4GlcNAcβ-Sp19	3	3	81
405	Galα1-4Galβ1-4GlcNAcβ1-2Manα1-6(Galα1-4Galβ1-4GlcNAcβ1-2Manα1-3)Manβ1-4GlcNAcβ1-4GlcNAcβ-Sp24	4	3	69
406	Galα1-3Galβ1-4GlcNAcβ1-3GalNAcα-Sp14	8	7	86
407	Galβ1-3GlcNAcβ1-6Galβ1-4GlcNAcβ-Sp0	5	3	60
408	Galβ1-3GlcNAcα1-6Galβ1-4GlcNAcβ-Sp0	5	5	117
409	GalNAcβ1-3Galα1-6Galβ1-4Glcβ-Sp8	3	4	132
410	Galα1-3(Fucα1-2)Galβ1-4(Fucα1-3)Glcβ-Sp21	9	6	73
411	Galβ1-4GlcNAcβ1-6(Neu5Acα2-6Galβ1-3GlcNAcβ1-3)Galβ1-4Glc-Sp21	5	3	48
412	Galβ1-3GalNAcβ1-4(Neu5Acα2-8Neu5Acα2-3)Galβ1-4Glcβ-Sp0	6	4	72
413	Neu5Acα2-3Galβ1-3GalNAcβ1-4(Neu5Acα2-8Neu5Acα2-3)Galβ1-4Glcβ-Sp0	8	2	29
414	Galα1-3(Fucα1-2)Galβ1-4GlcNAcβ1-3GalNAcα-Sp14	3	4	149
415	GalNAcα1-3(Fuca1-2)Galβ1-4GlcNAcβ1-3GalNAcα-Sp14	2	2	112
416	GalNAcα1-3GalNAcβ1-3Galα1-4Galβ1-4Glcβ-Sp0	1	3	247
417	Fucα1-2Galβ1-4(Fucα1-3)GlcNAcβ1-3GalNAcα-Sp14	1	4	369
418	Galα1-3(Fucα1-2)Galβ1-4(Fucα1-3)GlcNAcβ1-3GalNAc-Sp14	5	3	51
419	GalNAcα1-3(Fucα1-2)Galβ1-4(Fucα1-3)GlcNAcβ1-3GalNAc-Sp14	14	7	51
420	Galβ1-4(Fucα1-3)GlcNAcβ1-2Manα1-6(Galβ1-4(Fucα1-3)GlcNAcβ1-2Manα1-3)Manβ1-4GlcNAcβ1-4(Fucα1-6)GlcNAcβ-Sp22	11	3	26
421	Fucα1-2Galβ1-4GlcNAcβ1-2Manα1-6(Fucα1-2Galβ1-4GlcNAcβ1-2Manα1-3)Manβ1-4GlcNAcβ1-4(Fucα1-6)GlcNAcβ-Sp22	6	8	131
422	GlcNAcβ1-2(GlcNAcβ1-6)Manα1-6(GlcNAcβ1-2Manα1-3)Manβ1-4GlcNAcβ1-4GlcNAcβ-Sp19	12	8	64
423	Fucα1-2Galβ1-3GlcNAcβ1-3GalNAc-Sp14	2	6	340
424	Galα1-3(Fucα1-2)Galβ1-3GlcNAcβ1-3GalNAc-Sp14	4	3	71
425	GalNAcα1-3(Fucα1-2)Galβ1-3GlcNAcβ1-3GalNAc-Sp14	7	10	130
426	Galα1-3Galβ1-3GlcNAcβ1-3GalNAc-Sp14	20	30	151
427	Fucα1-2Galβ1-3GlcNAcβ1-2Manα1-6(Fucα1-2Galβ1-3GlcNAcβ1-2Manα1-3)Manβ1-4GlcNAcβ1-4(Fucα1-6)GlcNAcβ-Sp22	8	7	90
428	Galα1-3(Fucα1-2)Galβ1-4GlcNAcβ1-2Manα1-6(Galα1-3(Fucα1-2)Galβ1-4GlcNAcβ1-2Manα1-3)Manβ1-4GlcNAcβ1-4(Fucα1-6)GlcNAcβ-Sp22	3	2	81
429	Galβ1-3GlcNAcβ1-6(Galβ1-3GlcNAcβ1-2)Manα1-6(Galβ1-3GlcNAcβ1-2Manα1-3)Manβ1-4GlcNAcβ1-4GlcNAcβ-Sp19	8	4	52
430	Galβ1-4GlcNAcβ1-6(Fucα1-2Galβ1-3GlcNAcβ1-3)Galβ1-4Glc-Sp21	1	5	727
431	Fucα1-3GlcNAcβ1-6(Galβ1-4GlcNAcβ1-3)Galβ1-4Glc-Sp21	12	14	113
432	GlcNAcβ1-2Manα1-6(GlcNAcβ1-4)(GlcNAcβ1-2Manα1-3)Manβ1-4GlcNAcβ1-4GlcNAc-Sp21	9	2	18
433	GlcNAcβ1-2Manα1-6(GlcNAcβ1-4)(GlcNAcβ1-4(GlcNAcβ1-2)Manα1-3)Manβ1-4GlcNAcβ1-4GlcNAc-Sp21	4	3	94
434	GlcNAcβ1-6(GlcNAcβ1-2)Manα1-6(GlcNAcβ1-4)(GlcNAcβ1-2Manα1-3)Manβ1-4GlcNAcβ1-4GlcNAc-Sp21	6	6	112
435	GlcNAcβ1-6(GlcNAcβ1-2)Manα1-6(GlcNAcβ1-4)(GlcNAcβ1-4(GlcNAcβ1-2)Manα1-3)Manβ1-4GlcNAcβ1-4GlcNAc-Sp21	5	4	77

436	Galβ1-4GlcNAcβ1-2Manα1-6(GlcNAcβ1-4)(Galβ1-4GlcNAcβ1-2Manα1-3)Manβ1-4GlcNAcβ1-4GlcNAc-Sp21	12	7	59
437	Galβ1-4GlcNAcβ1-2Manα1-6(GlcNAcβ1-4)(Galβ1-4GlcNAcβ1-4(Galβ1-4GlcNAcβ1-2)Manα1-3)Manβ1-4GlcNAcβ1-4GlcNAc-Sp21	8	5	66
438	Galβ1-4GlcNAcβ1-6(Galβ1-4GlcNAcβ1-2)Manα1-6(GlcNAcβ1-4)(Galβ1-4GlcNAcβ1-2Manα1-3)Manβ1-4GlcNAcβ1-4GlcNAc-Sp21	2	6	267
439	Galβ1-4GlcNAcβ1-6(Galβ1-4GlcNAcβ1-2)Manα1-6(GlcNAcβ1-4)(Galβ1-4GlcNAcβ1-4(Galβ1-4GlcNAcβ1-2)Manα1-3)Manβ1-4GlcNAcβ1-4GlcNAc-Sp21	4	4	115
440	Galβ1-4Galβ-Sp10	4	4	90
441	Galβ1-6Galβ-Sp10	2	2	98
442	Neu5Acα2-3Galβ1-4GlcNAcβ1-3Galβ-Sp8	5	5	101
443	GalNAcβ1-6GalNAcβ-Sp8	6	5	85
444	(6S)Galβ1-3GlcNAcβ-Sp0	8	3	40
445	(6S)Galβ1-3(6S)GlcNAc-Sp0	10	4	45
446	Fucα1-2Galβ1-4 GlcNAcβ1-2Manα1-6(Fucα1-2Galβ1-4GlcNAcβ1-2(Fucα1-2Galβ1-4GlcNAcβ1-4)Manα1-3)Manβ1-4GlcNAcβ1-4GlcNAcβ-Sp12	4	4	98
447	Fucα1-2Galβ1-4(Fucα1-3)GlcNAcβ1-2Manα1-6(Fucα1-2Galβ1-4(Fucα1-3)GlcNAcβ1-4(Fucα1-2Galβ1-4(Fucα1-3)GlcNAcβ1-2)Manα1-3)Manβ1-4GlcNAcβ1-4GlcNAcβ-Sp12	5	7	138
448	Galβ1-4(Fucα1-3)GlcNAcβ1-6GalNAc-Sp14	16	10	63
449	Galβ1-4GlcNAcβ1-2Manα-Sp0	7	9	120
450	Fucα1-2Galβ1-4GlcNAcβ1-6(Fucα1-2Galβ1-4GlcNAcβ1-3)GalNAc-Sp14	6	3	46
451	Galα1-3Fucα1-2Galβ1-4GlcNAcβ1-6(Galα1-3Fucα1-2Galβ1-4GlcNAcβ1-3)GalNAc-Sp14	9	8	84
452	GalNAca1-3(Fucα1-2)Galβ1-4GlcNAcβ1-6(GalNAcα1-3(Fucα1-2)Galβ1-4GlcNAcβ1-3)GalNAc-Sp14	8	4	47
453	Neu5Acα2-8Neu5Acα2-3Galβ1-3GalNAcβ1-4(Neu5Acα2-8Neu5Acα2-3)Galβ1-4Glcβ-Sp0	11	5	51
454	GalNAcβ1-4Galβ1-4Glcβ-Sp0	7	1	16
455	GalNAcα1-3(Fucα1-2)Galβ1-4GlcNAcβ1-2Manα1-6(GalNAcα1-3(Fucα1-2)Galβ1-4GlcNAcβ1-2Manα1-3)Manβ1-4GlcNAcβ1-4(Fucα1-6)GlcNAcβ-Sp22	3	9	353
456	Galα1-3(Fucα1-2)Galβ1-3GlcNAcβ1-2Manα1-6(Galα1-3(Fucα1-2)Galβ1-3GlcNAcβ1-2Manα1-3)Manβ1-4GlcNAcβ1-4(Fucα1-6)GlcNAcβ-Sp22	7	5	75
457	Neu5Acα2-6Galβ1-4GlcNAcβ1-6(Fucα1-2Galβ1-3GlcNAcβ1-3)Galβ1-4Glc-Sp21	3	6	221
458	GalNAcα1-3(Fucα1-2)Galβ1-3GlcNAcβ1-2Manα1-6(GalNAcα1-3(Fucα1-2)Galβ1-3GlcNAcβ1-2Manα1-3)Manβ1-4GlcNAcβ1-4(Fucα1-6)GlcNAcβ-Sp22	8	12	139
459	Galβ1-4GlcNAcβ1-6(Galβ1-4GlcNAcβ1-2)Manα1-6(Galβ1-4GlcNAcβ1-2Manα1-3)Manβ1-4GlcNAcβ1-4GlcNAcβ-Sp19	6	5	92
460	Neu5Acα2-3Galβ1-4GlcNAcβ1-2Manα1-6(GlcNAcβ1-4)(Neu5Acα2-3Galβ1-4GlcNAcβ1-2Manα1-3)Manβ1-4GlcNAcβ1-4GlcNAcβ-Sp21	6	2	33
461	Neu5Acα2-3Galβ1-4GlcNAcβ1-4Manα1-6(GlcNAcβ1-4)(Neu5Acα2-3Galβ1-4GlcNAcβ1-4(Neu5Acα2-3Galβ1-4GlcNAcβ1-2)Manα1-3)Manβ1-4GlcNAcβ1-4GlcNAcβ-Sp21	12	7	55
462	Neu5Acα2-3Galβ1-4GlcNAcβ1-6(Neu5Acα2-3Galβ1-4GlcNAcβ1-2)Manα1-6(GlcNAcβ1-4)(Neu5Acα2-3Galβ1-4GlcNAcβ1-2Manα1-3)Manβ1-4GlcNAcβ1-4GlcNAcβ-Sp21	4	3	93
463	Neu5Acα2-3Galβ1-4GlcNAcβ1-6(Neu5Acα2-3Galβ1-4GlcNAcβ1-2)Manα1-6(GlcNAcβ1-4)(Neu5Acα2-3Galβ1-4GlcNAcβ1-4(Neu5Acα2-3Galβ1-4GlcNAcβ1-2)Manα1-3)Manβ1-4GlcNAcβ1-4GlcNAcβ-Sp21	7	7	110
464	Neu5Acα2-6Galβ1-4GlcNAcβ1-2Manα1-6(GlcNAcβ1-4)(Neu5Acα2-6Galβ1-4GlcNAcβ1-2Manα1-3)Manβ1-4GlcNAcβ1-4GlcNAcβ-Sp21	21	38	185
465	Neu5Acα2-6Galβ1-4GlcNAcβ1-4Manα1-6(GlcNAcβ1-4)(Neu5Acα2-6Galβ1-4GlcNAcβ1-4(Neu5Acα2-6Galβ1-4GlcNAcβ1-2)Manα1-3)Manβ1-4GlcNAcβ1-4GlcNAcβ-Sp21	15	12	82
466	Neu5Acα2-6Galβ1-4GlcNAcβ1-6(Neu5Acα2-6Galβ1-4GlcNAcβ1-2)Manα1-6(GlcNAcβ1-4)(Neu5Acα2-6Galβ1-4GlcNAcβ1-2Manα1-3)Manβ1-4GlcNAcβ1-4GlcNAcβ-Sp21	1	2	191
467	Neu5Acα2-6Galβ1-4GlcNAcβ1-6(Neu5Acα2-6Galβ1-4GlcNAcβ1-2)Manα1-6(GlcNAcβ1-4)(Neu5Acα2-6Galβ1-4GlcNAcβ1-4(Neu5Acα2-6Galβ1-4GlcNAcβ1-2)Manα1-3)Manβ1-4GlcNAcβ1-4GlcNAcβ-Sp21	10	11	117
468	Galα1-3(Fucα1-2)Galβ1-3GalNAcα-Sp8	5	2	44

469	Galα1-3(Fucα1-2)Galβ1-3GalNAcβ-Sp8	18	7	39
470	Glcα1-6Glcα1-6Glcα1-6Glcβ-Sp10	8	3	43
471	Glcα1-4Glcα1-4Glcα1-4Glcβ-Sp10	15	9	58
472	Neu5Acα2-3Galβ1-4GlcNAcβ1-6(Neu5Acα2-3Galβ1-4GlcNAcβ1-3)GalNAcα-Sp14	3	3	97
473	Fucα1-2Galβ1-4(Fucα1-3)GlcNAcβ1-2Manα1-6(Fucα1-2Galβ1-4(Fucα1-3)GlcNAcβ1-2Manα1-3)Manβ1-4GlcNAcβ1-4(Fucα1-6)GlcNAcβ-Sp24	10	6	64
474	Fucα1-2Galβ1-3(Fucα1-4)GlcNAcβ1-2Manα1-6(Fucα1-2Galβ1-3(Fucα1-4)GlcNAcβ1-2Manα1-3)Manβ1-4GlcNAcβ1-4(Fucα1-6)GlcNAcβ1-4(Fucα1-6)GlcNAcβ-Sp19	9	4	44
475	Neu5Acα2-3Galβ1-3GlcNAcβ1-6(Neu5Acα2-3Galβ1-4GlcNAcβ1-2)Manα1-6(Neu5Acα2-3Galβ1-3GlcNAcβ1-2Manα1-3)Manβ1-4GlcNAcβ1-4GlcNAcβ-Sp19	1	6	906
476	GlcNAcβ1-6(GlcNAcβ1-2)Manα1-6(GlcNAcβ1-2Manα1-3)Manβ1-4GlcNAcβ1-4(Fucα1-6)GlcNAcβ-Sp24	7	6	92
477	Galβ1-3GlcNAcβ1-2Manα1-6(GlcNAcβ1-4)(Galβ1-3GlcNAcβ1-2Manα1-3)Manβ1-4GlcNAcβ1-4GlcNAcβ-Sp21	7	12	169
478	Neu5Acα2-6Galβ1-4GlcNAcβ1-6(Galβ1-3GlcNAcβ1-3)Galβ1-4Glcβ-Sp21	10	4	38
479	Neu5Acα2-3Galβ1-4GlcNAcβ1-2Manα-Sp0	9	5	57
480	Neu5Acα2-3Galβ1-4GlcNAcβ1-6GalNAcα-Sp14	8	6	72
481	Neu5Acα2-6Galβ1-4GlcNAcβ1-6GalNAcα-Sp14	5	4	79
482	Neu5Acα2-6Galβ1-4 GlcNAcβ1-6(Neu5Acα2-6Galβ1-4GlcNAcβ1-3)GalNAcα-Sp14	4	2	55
483	Neu5Acα2-6Galβ1-4GlcNAcβ1-2Manα1-6(Neu5Acα2-6Galβ1-4GlcNAcβ1-2Manα1-3)Manβ1-4GlcNAcβ1-4(Fucα1-6)GlcNAcβ-Sp24	8	5	65
484	Neu5Acα2-3Galβ1-4GlcNAcβ1-2Manα1-6(Neu5Acα2-3Galβ1-4GlcNAcβ1-2Manα1-3)Manβ1-4GlcNAcβ1-4(Fucα1-6)GlcNAcβ-Sp24	7	3	41
485	Manα1-6(Manα1-3)Manβ1-4GlcNAcβ1-4(Fucα1-6)GlcNAcβ-Sp19	-1	1	-143
486	Galβ1-4GlcNAcβ1-6(Galβ1-4GlcNAcβ1-2)Manα1-6(Galβ1-4GlcNAcβ1-2Manα1-3)Manβ1-4GlcNAcβ1-4(Fucα1-6)GlcNAcβ-Sp24	2	1	47
487	Neu5Acα2-3Galβ1-3GlcNAcβ1-2Manα1-6(GlcNAcβ1-4)(Neu5Acα2-3Galβ1-3GlcNAcβ1-2Manα1-3)Manβ1-4GlcNAcβ1-4GlcNAc-Sp21	4	1	25
488	Neu5Acα2-6Galβ1-4GlcNAcβ1-6(Fucα1-2Galβ1-4(Fucα1-3)GlcNAcβ1-3)Galβ1-4Glc-Sp21	13	12	97
489	Galβ1-3GlcNAcβ1-6GalNAcα-Sp14	4	2	59
490	Galα1-3Galβ1-3GlcNAcβ1-6GalNAcα-Sp14	8	3	38
491	Galβ1-3(Fucα1-4)GlcNAcβ1-6GalNAcα-Sp14	7	5	68
492	Neu5Acα2-3Galβ1-3GlcNAcβ1-6GalNAcα-Sp14	4	7	190
493	(3S)Galβ1-3(Fucα1-4)GlcNAcβ-Sp0	4	2	39
494	Galβ1-4(Fucα1-3)GlcNAcβ1-6(Neu5Acα2-6(Neu5Acα2-3Galβ1-3)GlcNAcβ1-3)Galb1-4Glc-Sp21	-5	2	-42
495	Fucα1-2Galβ1-4GlcNAcβ1-6GalNAcα-Sp14	7	8	116
496	Galα1-3Galβ1-4GlcNAcβ1-6GalNAcα-Sp14	6	6	101
497	Galβ1-4(Fucα1-3)GlcNAcβ1-2Manα-Sp0	5	1	11
498	Fucα1-2(6S)Galβ1-3GlcNAcβ-Sp0	4	2	66
499	Galα1-3(Fucα1-2)Galβ1-4GlcNAcβ1-6GalNAcα-Sp14	5	3	56
500	Fucα1-2Galβ1-4GlcNAcβ1-2Manα-Sp0	19	10	53
501	Fucα1-2Galβ1-3(6S)GlcNAcβ-Sp0	10	5	51
502	Fucα1-2(6S)Galβ1-3(6S)GlcNAcβ-Sp0	8	5	63
503	Neu5Acα2-6GalNAcβ1-4(6S)GlcNAcβ-Sp8	7	11	155
504	GalNAcβ1-4(Fucα1-3)(6S)GlcNAcβ-Sp8	3	8	249
505	(3S)GalNAcβ1-4(Fucα1-3)GlcNAcβ-Sp8	10	6	63
506	Fucα1-2Galβ1-3GlcNAcβ1-6(Fucα1-2Galβ1-3GlcNAcβ1-3)GalNAcα-Sp14	4	1	21
507	GalNAcα1-3(Fucα1-2)Galβ1-3GlcNAcβ1-6GalNAcα-Sp14	10	6	56
508	GlcNAcβ1-6(GlcNAcβ1-2)Manα1-6(GlcNAcβ1-4)(GlcNAcβ1-4(GlcNAcβ1-2)Manα1-3)Manβ1-4GlcNAcβ1-4(Fucα1-6)GlcNAc-Sp21	6	5	91
509	Galβ1-4GlcNAcβ1-6(Galβ1-4GlcNAcβ1-2)Manα1-6(GlcNAcβ1-4)Galβ1-4GlcNAcβ1-4(Galβ1-4GlcNAcβ1-2)Manα1-3)Manβ1-4GlcNAcβ1-4(Fucα1-6)GlcNAc-Sp21	3	3	102

510	Galβ1-3GlcNAcα1-3Galβ1-4GlcNAcβ-Sp8	3	3	114
511	Galβ1-3(6S)GlcNAcβ-Sp8	3	2	60
512	(6S)(4S)GalNAcβ1-4GlcNAc-Sp8	1	2	163
513	(6S)GalNAcβ1-4GlcNAc-Sp8	2	2	73
514	(3S)GalNAcβ1-4(3S)GlcNAc-Sp8	11	8	74
515	GalNAcβ1-4(6S)GlcNAc-Sp8	10	13	133
516	(3S)GalNAcβ1-4GlcNAc-Sp8	7	2	31
517	(4S)GalNAcβ-Sp10	4	2	48
518	Galβ1-4(6P)GlcNAcβ-Sp0	3	3	101
519	(6P)Galβ1-4GlcNAcβ-SP0	7	4	60
520	GalNAcα1-3(Fucα1-2)Galβ1-4GlcNAcβ1-6GalNAc-Sp14	8	4	54
521	Neu5Acα2-6Galβ1-4GlcNAcβ1-2Man-Sp0	2	4	171
522	Galα1-3Galβ1-4GlcNAcβ1-2Manα-Sp0	7	3	41
523	Galα1-3(Fucα1-2)Galβ1-4GlcNAcβ1-2Manα-Sp0	7	4	63
524	GalNAcα1-3(Fucα1-2)Galβ1-4 GlcNAcβ1-2Manα-Sp0	6	4	74
525	Galβ1-3GlcNAcβ1-2Manα-Sp0	13	14	106
526	Galα1-3(Fucα1-2)Galβ1-3GlcNAcβ1-6GalNAc-Sp14	3	3	92
527	Neu5Acα2-3Galβ1-3GlcNAcβ1-2Manα-Sp0	4	4	105
528	Galα1-3Galβ1-3GlcNAcβ1-2Manα-Sp0	7	8	115
529	GalNAcβ1-4GlcNAcβ1-2Manα-Sp0	27	47	175
530	Neu5Acα2-3Galβ1-3GlcNAcβ1-4Galβ1-4Glcβ-Sp0	0	2	373
531	GlcNAcβ1-2 Manα1-6(GlcNAcβ1-4)(GlcNAcβ1-2Manα1-3)Manβ1-4GlcNAcβ1-4(Fucα1-6)GlcNAc-Sp21	5	3	66
532	Galβ1-4GlcNAcβ1-2 Manα1-6(GlcNAcβ1-4)(Galβ1-4GlcNAcβ1-2Manα1-3)Manβ1-4GlcNAcβ1-4(Fucα1-6)GlcNAc-Sp21	10	5	57
533	Galβ1-4GlcNAcβ1-2 Manα1-6(Galβ1-4GlcNAcβ1-4)(Galβ1-4GlcNAcβ1-2Manα1-3)Manβ1-4GlcNAcβ1-4(Fucα1-6)GlcNAc-Sp21	8	6	68
534	Fucα1-4(Galβ1-3)GlcNAcβ1-2 Manα-Sp0	3	4	130
535	Neu5Acα2-3Galβ1-4(Fucα1-3)GlcNAcβ1-2Manα-Sp0	14	5	33
536	GlcNAcβ1-3Galβ1-4GlcNAcβ1-6(GlcNAcβ1-3)Galβ1-4GlcNAc-Sp0	0	5	-1332
537	GalNAcα1-3(Fucα1-2)Galβ1-3GalNAcβ1-3Galα1-4Galβ1-4Glc-Sp21	7	9	138
538	Galα1-3(Fucα1-2)Galβ1-3GalNAcβ1-3Galα1-4Galβ1-4Glc-Sp21	8	7	87
539	Galβ1-3GalNAcβ1-3Gal-Sp21	8	4	46
540	GlcNAcβ1-3Galβ1-4GlcNAcβ1-2Manα1-6(GlcNAcβ1-3Galβ1-4GlcNAcβ1-2Manα1-3)Manβ1-4GlcNAcβ1-4GlcNAcβ-Sp12	3	3	115
541	GlcNAcβ1-3Galβ1-4GlcNAcβ1-2Manα1-6(GlcNAcβ1-3Galβ1-4GlcNAcβ1-2Manα1-3)Manβ1-4GlcNAcβ1-4GlcNAcβ-Sp25	6	5	90
542	Galβ1-4GlcNAcβ1-3Galβ1-4GlcNAcβ1-2Manα1-6(Galβ1-4GlcNAcβ1-3Galβ1-4GlcNAcβ1-2Manα1-3)Manβ1-4GlcNAcβ1-4GlcNAcβ-Sp12	5	3	51
543	Galβ1-4GlcNAcβ1-3Galβ1-4GlcNAcβ1-2Manα1-6(Galβ1-4GlcNAcβ1-3Galβ1-4GlcNAcβ1-2Manα1-3)Manβ1-4GlcNAcβ1-4GlcNAcβ-Sp24	4	4	111
544	Neu5Gcα2-3Galβ1-4GlcNAcβ1-3Galβ1-4GlcNAcβ1-2Manα1-6(Neu5Gcα2-3Galβ1-4GlcNAcβ1-3Galβ1-4GlcNAcβ1-2Manα1-3)Manβ1-4GlcNAcβ1-4GlcNAcβ-Sp24	5	6	125
545	Fucα1-2Galβ1-4GlcNAcβ1-3Galβ1-4GlcNAcβ1-2Manα1-6(Fucα1-2Galβ1-4GlcNAcβ1-3Galβ1-4GlcNAcβ1-2Manα1-3)Manβ1-4GlcNAcβ1-4GlcNAcβ-Sp24	4	6	160
546	GlcNAcβ1-3Galβ1-4GlcNAcβ1-3Galβ1-4GlcNAcβ1-2Manα1-6(GlcNAcβ1-3Galβ1-4GlcNAcβ1-3Galβ1-4GlcNAcβ1-2Manα1-3)Manβ1-4GlcNAcβ1-4GlcNAcβ-Sp12	7	6	94
547	GlcNAcβ1-3Galβ1-4GlcNAcβ1-3Galβ1-4GlcNAcβ1-2Manα1-6(GlcNAcβ1-3Galβ1-4GlcNAcβ1-3Galβ1-4GlcNAcβ1-2Manα1-3)Manβ1-4GlcNAcβ1-4GlcNAcβ-Sp25	6	4	70
548	Galβ1-4GlcNAcβ1-3Galβ1-4GlcNAcβ1-3Galβ1-4GlcNAcβ1-2Manα1-6(Galβ1-4GlcNAcβ1-3Galβ1-4GlcNAcβ1-3Galβ1-4GlcNAcβ1-2Manα1-3)Manβ1-4GlcNAcβ1-4GlcNAcβ-Sp12	15	11	69
549	Galβ1-4GlcNAcβ1-3Galβ1-4GlcNAcβ1-3Galβ1-4GlcNAcβ1-2Manα1-6(Galβ1-4GlcNAcβ1-3Galβ1-4GlcNAcβ1-3Galβ1-4GlcNAcβ1-2Manα1-3)Manβ1-4GlcNAcβ1-4GlcNAcβ-Sp24	9	9	95

550	GlcNAcβ1-3Galβ1-4GlcNAcβ1-3Galβ1-4GlcNAcβ1-3Galβ1-4GlcNAcβ1-2Manα1-6(GlcNAcβ1-3Galβ1-4GlcNAcβ1-3Galβ1-4GlcNAcβ1-3Galβ1-4GlcNAcβ1-2Manα1-3)Manβ1-4GlcNAcβ1-4GlcNAcβ-Sp25	13	2	16
551	Galβ1-4GlcNAcβ1-3Galβ1-4GlcNAcβ1-3Galβ1-4GlcNAcβ1-3Galβ1-4GlcNAcβ1-2Manα1-6(Galβ1-4GlcNAcβ1-3Galβ1-4GlcNAcβ1-3Galβ1-4GlcNAcβ1-3Galβ1-4GlcNAcβ1-2Manα1-3)Manβ1-4GlcNAcβ1-4GlcNAcβ-Sp25	50	58	115
552	Galβ1-3GlcNAcβ1-3Galβ1-4GlcNAcβ1-2Manα1-6(Galβ1-3GlcNAcβ1-3Galβ1-4GlcNAcβ1-2Manα1-3)Manβ1-4GlcNAcβ1-4GlcNAc-Sp25	4	6	144
553	Neu5Gcα2-8Neu5Gcα2-3Galβ1-4GlcNAc-Sp0	2	3	152
554	Neu5Acα2-8Neu5Gcα2-3Galβ1-4GlcNAc-Sp0	3	4	119
555	Neu5Gcα2-8Neu5Acα2-3Galβ1-4GlcNAc-Sp0	3	4	123
556	Neu5Gcα2-8Neu5Gcα2-3Galβ1-4GlcNAcβ1-3Galβ1-4GlcNAc-Sp0	9	4	40
557	Neu5Gcα2-8Neu5Gcα2-6Galβ1-4GlcNAc-Sp0	2	1	31
558	Neu5Acα2-8Neu5Acα2-3Galβ1-4GlcNAc-Sp0	14	12	83
559	GlcNAcβ1-3Galβ1-4GlcNAcβ1-6(GlcNAcβ1-3Galβ1-4GlcNAcβ1-2)Manα1-6(GlcNAcβ1-3Galβ1-4GlcNAcβ1-2Manα1-3)Manβ1-4GlcNAcβ1-4GlcNAc-Sp24	6	2	28
560	Galβ1-4GlcNAcβ1-3Galβ1-4GlcNAcβ1-6(Galβ1-4GlcNAcβ1-3Galβ1-4GlcNAcβ1-2)Manα1-6(Galβ1-4GlcNAcβ1-3Galβ1-4GlcNAcβ1-2Manα1-3)Manα1-4GlcNAcβ1-4GlcNAc-Sp24	15	8	55
561	Galα1-3Galβ1-4GlcNAcβ1-2Manα1-6(Galα1-3Galβ1-4GlcNAcβ1-2Manα1-3)Manβ1-4GlcNAcβ1-4GlcNAc-Sp24	8	4	53
562	GlcNAcβ1-3Galβ1-4GlcNAcβ1-6(GlcNAcβ1-3Galβ1-3)GalNAcα-Sp14	5	2	53
563	GalNAcβ1-3GlcNAcβ-Sp0	12	5	44
564	GalNAcβ1-4GlcNAcβ1-3GalNAcβ1-4GlcNAcβ-Sp0	4	5	127
565	GlcNAcβ1-3Galβ1-4GlcNAcβ1-3Galβ1-4GlcNAcβ1-3Galβ1-4GlcNAcβ1-3Galβ1-4GlcNAcβ1-2Manα1-6(GlcNAcβ1-3Galβ1-4GlcNAcβ1-3Galβ1-4GlcNAcβ1-3Galβ1-4GlcNAcβ1-3Galβ1-4GlcNAcβ1-2Manα1-3)Manβ1-4GlcNAcβ1-4GlcNAcβ-Sp25	16	10	66
566	Galβ1-4GlcNAcβ1-3Galβ1-4GlcNAcβ1-3Galβ1-4GlcNAcβ1-3Galβ1-4GlcNAcβ1-3Galβ1-4GlcNAcβ1-2Manα1-6(Galβ1-4GlcNAcβ1-3Galβ1-4GlcNAcβ1-3Galβ1-4GlcNAcβ1-3Galβ1-4GlcNAcβ1-3Galβ1-4GlcNAcβ1-2Manα1-3)Manβ1-4GlcNAcβ1-4GlcNAcβ-Sp25	8	5	61
567	GlcNAβ1-3Galβ1-3GalNAc-Sp14	16	4	23
568	Galβ1-3GlcNAcβ1-6(Galβ1-3)GalNAc-Sp14	10	4	41
569	Galβ1-4GlcNAcβ1-3Galβ1-4GlcNAcβ1-3Galβ1-4GlcNAcβ1-3Galβ1-4GlcNAcβ1-3Galβ1-4GlcNAcβ1-3Galβ1-4GlcNAcβ1-2Manα1-6(Galβ1-4GlcNAcβ1-3Galβ1-4GlcNAcβ1-3Galβ1-4GlcNAcβ1-3Galβ1-4GlcNAcβ1-3Galβ1-4GlcNAcβ1-3Galβ1-4GlcNAcβ1-2Manα1-3)Manβ1-4GlcNAcβ1-4GlcNAcβ-Sp25	8	5	63
570	(3S)GlcAβ1-3Galβ1-4GlcNAcβ1-3Galβ1-4Glc-Sp0	0	3	-1282
571	(3S)GlcAβ1-3Galβ1-4GlcNAcβ1-2Manα-Sp0	4	1	42
572	Galβ1-3GlcNAcβ1-3Galβ1-4GlcNAcβ1-3Galβ1-4GlcNAcβ1-6(Galβ1-3GlcNAcβ1-3Galβ1-4GlcNAcβ1-3Galβ1-4GlcNAβ1-2)Manα1-6(Galβ1-3GlcNAcβ1-3Galβ1-4GlcNAcβ1-3Galβ1-4GlcNAcβ1-2Manα1-3)Manβ1-4GlcNAcβ1-4(Fucα1-6)GlcNAcβ-Sp24	3	2	82
573	Galβ1-3GlcNAcβ1-3Galβ1-4GlcNAcβ1-6(Galβ1-3GlcNAcβ1-3Galβ1-4GlcNAβ1-2)Manα1-6(Galβ1-3GlcNAcβ1-3Galβ1-4GlcNAcβ1-2Manα1-3)Manβ1-4GlcNAcβ1-4(Fucα1-6)GlcNAcβ-Sp24	6	1	21
574	Neu5Acα2-8Neu5Acα2-3Galβ1-3GalNAcβ1-4(Neu5Acα2-3)Galβ1-4Glc-Sp21	2	4	225
575	GlcNAcβ1-3Galβ1-4GlcNAcβ1-2Manα1-6(GlcNAcβ1-3Galβ1-4GlcNAcβ1-2Manα1-3)Manβ1-4GlcNAcβ1-4(Fucα1-6)GlcNAcβ-Sp24	3	8	235
576	Galβ1-4GlcNAcβ1-3Galβ1-4GlcNAcβ1-2Manα1-6(Galβ1-4GlcNAcβ1-3Galβ1-4GlcNAcβ1-2Manα1-3)Manβ1-4GlcNAcβ1-4(Fucα1-6)GlcNAcβ-Sp24	8	9	106
577	GlcNAcβ1-3Galβ1-4GlcNAcβ1-3Galβ1-4GlcNAcβ1-2Manα1-6(GlcNAcβ1-3Galb1-4GlcNAcβ1-3Galβ1-4GlcNAcβ1-2Manα1-3)Manβ1-4GlcNAcβ1-4(Fucα1-6)GlcNAcβ-Sp24	7	14	194
578	Galβ1-4GlcNAcβ1-3Galβ1-4GlcNAcβ1-3Galβ1-4GlcNAcβ1-2Manα1-6(Galβ1-4GlcNAcβ1-3Galβ1-4GlcNAcβ1-3Galβ1-4GlcNAcβ1-2Manα1-3)Manβ1-4GlcNAcβ1-4(Fucα1-6)GlcNAcβ-Sp24	12	5	43
579	GlcNAcβ1-3Galβ1-4GlcNAcβ1-3Galβ1-4GlcNAcβ1-3Galβ1-4GlcNAcβ1-2Manα1-6(GlcNAcβ1-3Galβ1-4GlcNAcβ1-3Galβ1-4GlcNAcβ1-3Galβ1-4GlcNAcβ1-2Manα1-3)Manβ1-4GlcNAcβ1-4(Fucα1-6)GlcNAcβ-Sp24	14	7	48

580	Galβ1-4GlcNAcβ1-3Galβ1-4GlcNAcβ1-3Galβ1-4GlcNAcβ1-3Galβ1-4GlcNAcβ1-2Manα1-6(Galβ1-4GlcNAcβ1-3Galβ1-4GlcNAcβ1-3Galβ1-4GlcNAcβ1-3Galβ1-4GlcNAcβ1-2Manα1-3)Manβ1-4GlcNAcβ1-4(Fucα1-6)GlcNAcβ-Sp24	26	16	65
581	GlcNAcβ1-3Galβ1-4GlcNAcβ1-3Galβ1-4GlcNAcβ1-3Galβ1-4GlcNAcβ1-3Galβ1-4GlcNAcβ1-2Manα1-6(GlcNAcβ1-3Galβ1-4GlcNAcβ1-3Galβ1-4GlcNAcβ1-3Galβ1-4GlcNAcβ1-3Galβ1-4GlcNAcβ1-2Manα1-3)Manβ1-4GlcNAcβ1-4(Fucα1-6)GlcNAcβ-Sp19	21	10	50
582	Galβ1-4GlcNAcβ1-3Galβ1-4GlcNAcβ1-3Galβ1-4GlcNAcβ1-3Galβ1-4GlcNAcβ1-3Galβ1-4GlcNAcβ1-2Manα1-6(Galβ1-4GlcNAcβ1-3Galβ1-4GlcNAcβ1-3Galβ1-4GlcNAcβ1-3Galβ1-4GlcNAcβ1-3Galβ1-4GlcNAcβ1-2Manα1-3)Manβ1-4GlcNAcβ1-4(Fucα1-6)GlcNAcβ-Sp19	21	6	30
583	Galβ1-4GlcNAcβ1-3Galβ1-4GlcNAcβ1-6(Galβ1-4GlcNAcβ1-3Galβ1-4GlcNAβ1-2)Manα1-6(Galβ1-4GlcNAcβ1-3Galβ1-4GlcNAcβ1-2Manα1-3)Manβ1-4GlcNAcβ1-4(Fucα1-6)GlcNAcβ-Sp24	9	7	80
584	GlcNAcβ1-3Galβ1-4GlcNAcβ1-3Galβ1-4GlcNAcβ1-6(GlcNAcβ1-3Galβ1-4GlcNAcβ1-3Galβ1-4GlcNAβ1-2)Manα1-6(GlcNAcβ1-3Galβ1-4GlcNAcβ1-3Galβ1-4GlcNAcβ1-2Manα1-3)Manβ1-4GlcNAcβ1-4(Fucα1-6)GlcNAcβ-Sp24	5	1	12
585	Galβ1-4GlcNAcβ1-3Galβ1-4GlcNAcβ1-3Galβ1-4GlcNAcβ1-6(Galβ1-4GlcNAcβ1-3Galβ1-4GlcNAcβ1-3Galβ1-4GlcNAβ1-2)Manα1-6(Galβ1-4GlcNAcβ1-3Galβ1-4GlcNAcβ1-3Galβ1-4GlcNAcβ1-2Manα1-3)Manβ1-4GlcNAcβ1-4(Fucα1-6)GlcNAcβ-Sp24	10	5	49
586	GlcNAcβ1-3Galβ1-4GlcNAcβ1-3Galβ1-4GlcNAcβ1-3Galβ1-4GlcNAcβ1-6(GlcNAcβ1-3Galβ1-4GlcNAcβ1-3Galβ1-4GlcNAcβ1-3Galβ1-4GlcNAβ1-2)Manα1-6(GlcNAcβ1-3Galβ1-4GlcNAcβ1-3Galβ1-4GlcNAcβ1-3Galβ1-4GlcNAcβ1-2Manα1-3)Manβ1-4GlcNAcβ1-4(Fucα1-6)GlcNAcβ-Sp24	8	3	37
587	Galβ1-4GlcNAcβ1-3Galβ1-4GlcNAcβ1-3Galβ1-4GlcNAcβ1-3Galβ1-4GlcNAcβ1-6(Galβ1-4GlcNAcβ1-3Galβ1-4GlcNAcβ1-3Galβ1-4GlcNAcβ1-3Galβ1-4GlcNAβ1-2)Manα1-6(Galβ1-4GlcNAcβ1-3Galβ1-4GlcNAcβ1-3Galβ1-4GlcNAcβ1-3Galβ1-4GlcNAcβ1-2Manα1-3)Manβ1-4GlcNAcβ1-4(Fucα1-6)GlcNAcβ-Sp24	20	4	21
588	GlcNAcβ1-3Galβ1-4GlcNAcβ1-3Galβ1-4GlcNAcβ1-3Galβ1-4GlcNAcβ1-3Galβ1-4GlcNAcβ1-6(GlcNAcβ1-3Galβ1-4GlcNAcβ1-3Galβ1-4GlcNAcβ1-3Galβ1-4GlcNAcβ1-3Galβ1-4GlcNAβ1-2)Manα1-6(GlcNAcβ1-3Galβ1-4GlcNAcβ1-3Galβ1-4GlcNAcβ1-3Galβ1-4GlcNAcβ1-3Galβ1-4GlcNAcβ1-2Manα1-3)Manβ1-4GlcNAcβ1-4(Fucα1-6)GlcNAcβ-Sp24	3	4	111
589	Galβ1-4GlcNAcβ1-3Galβ1-4GlcNAcβ1-3Galβ1-4GlcNAcβ1-3Galβ1-4GlcNAcβ1-3Galβ1-4GlcNAcβ1-6(Galβ1-4GlcNAcβ1-3Galβ1-4GlcNAcβ1-3Galβ1-4GlcNAcβ1-3Galβ1-4GlcNAcβ1-3Galβ1-4GlcNAβ1-2)Manα1-6(Galβ1-4GlcNAcβ1-3Galβ1-4GlcNAcβ1-3Galβ1-4GlcNAcβ1-3Galβ1-4GlcNAcβ1-3Galβ1-4GlcNAcβ1-2Manα1-3)Manβ1-4GlcNAcβ1-4(Fucα1-6)GlcNAcβ-Sp24	13	9	67
590	Galβ1-4GlcNAcβ1-3Galβ1-4GlcNAcβ1-3GalNAcα-Sp14	5	3	58
591	Galβ1-4GlcNAcβ1-3Galβ1-4GlcNAcβ1-6(Galβ1-3)GalNAcα-Sp14	9	3	37
592	Galβ1-4GlcNAcβ1-3Galβ1-4GlcNAcβ1-6(Galβ1-4GlcNAcβ1-3Galβ1-4GlcNAcβ1-3)GalNAcα-Sp14	14	7	52
593	Neu5Acα2-3Galβ1-4GlcNAcβ1-3Galβ1-4GlcNAcβ1-3GalNAcα-Sp14	2	3	131
594	GlcNAcβ1-3Galβ1-4GlcNAcβ1-3GalNAcα-Sp14	6	8	128
595	GlcNAcβ1-3Galβ1-4GlcNAcβ1-6(Galβ1-3)GalNAcα-Sp14	14	11	81
596	GlcNAcβ1-3Galβ1-4GlcNAcβ1-6(GlcNAcβ1-3Galβ1-4GlcNAcβ1-3)GalNAcα-Sp14	5	1	29
597	Neu5Acα2-3Galβ1-4GlcNAcβ1-3Galβ1-4GlcNAcβ1-6(Neu5Acα2-3Galβ1-4GlcNAcβ1-3Galβ1-4GlcNAcβ1-3)GalNAcα-Sp14	10	6	53
598	Neu5Acα2-6Galβ1-4GlcNAcβ1-3Galβ1-4GlcNAcβ1-3GalNAcα-Sp14	6	7	131
599	GlcNAcβ1-3Galβ1-4GlcNAcβ1-3Galβ1-4GlcNAcβ1-3GalNAcα-Sp14	9	7	84
600	Galβ1-4GlcNAcβ1-3Galβ1-3GalNAcα-Sp14	11	12	111
601	Neu5Acα2-3Galβ1-4GlcNAcβ1-3Galβ1-4GlcNAcβ1-6(Galβ1-3)GalNAcα-Sp14	14	17	121
602	Neu5Acα2-6Galβ1-4GlcNAcβ1-3Galβ1-4GlcNAcβ1-6(Galβ1-3)GalNAcα-Sp14	2	2	96
603	Neu5Acα2-6Galβ1-4GlcNAcβ1-6(Galβ1-3)GalNAcα-Sp14	18	6	32
604	Neu5Acα2-3Galβ1-4GlcNAcβ1-3Galβ1-4GlcNAcβ1-2Manα1-6(Neu5Acα2-3Galβ1-4GlcNAcβ1-3Galβ1-4GlcNAcβ1-2Manα1-3)Manβ1-4GlcNAcβ1-4GlcNAcβ-Sp12	6	2	38
605	GlcNAcβ1-6(Neu5Acα2-3Galβ1-3)GalNAcα-Sp14	16	12	75
606	Neu5Acα2-6Galβ1-4GlcNAcβ1-3Galβ1-4GlcNAcβ1-6(Neu5Acα2-6Galβ1-4GlcNAcβ1-3Galβ1-4GlcNAcβ1-3)GalNAcα-Sp14	19	5	29

607	Neu5Acα2-6Galβ1-4GlcNAcβ1-3Galβ1-4GlcNAcβ1-3Galβ1-4GlcNAcβ1-2Manα1-6(Neu5Acα2-6Galβ1-4GlcNAcβ1-3Galβ1-4GlcNAcβ1-3Galβ1-4GlcNAcβ1-2Manα1-3)Manβ1-4GlcNAcβ1-4GlcNAcβ-Sp12	13	2	16
608	Neu5Acα2-3Galβ1-4GlcNAcβ1-3Galβ1-4GlcNAcβ1-3Galβ1-4GlcNAcβ1-2Manα1-6(Neu5Acα2-3Galβ1-4GlcNAcβ1-3Galβ1-4GlcNAcβ1-3Galβ1-4GlcNAcβ1-2Manα1-3)Manβ1-4GlcNAcβ1-4GlcNAcβ-Sp12	19	4	24
609	Neu5Acα2-6Galβ1-4GlcNAcβ1-3Galβ1-4GlcNAcβ1-2Manα1-6(Neu5Acα2-6Galβ1-4GlcNAcβ1-3Galβ1-4GlcNAcβ1-2Manα1-3)Manβ1-4GlcNAcβ1-4GlcNAcβ-Sp12	12	8	64
610	GlcNAcβ1-3Fucα-Sp21	2	3	137
611	Galβ1-3GalNAcβ1-4(Neu5Acα2-8Neu5Acα2-8Neu5Acα2-3)Galβ1-4Glcβ-Sp21	8	7	81

[a]Average relative fluorescence of six replicates.
[b]Standard deviation.
[c]Coefficient of variation expressed as %.
[d]Spacer arm code:
Sp0, $CH_2CH_2NH_2$; Sp8, $CH_2CH_2CH_2NH_2$; Sp9, $CH_2CH_2CH_2CH_2CH_2NH_2$; Sp10, $NHCOCH_2NH$; Sp11, $OCH_2C_6H_4$-p-$NHCOCH_2NH$; Sp12, Asparagine; Sp13, Glycine; Sp14, Threonine; Sp15, Serine; Sp16, PNP ($OC_6H_4NH_2$); Sp17, $OCH_2C_6H_4NH_2$; Sp18, $O(CH_2)_3NHCO(CH_2)_5NH_2$; Sp19, EN or NK; Sp20, GENR; Sp21, $N(CH_3)$-O-$(CH_2)_2$-NH_2; Sp22, NST; Sp23, $(OCH_2CH_2)_6NH_2$; Sp24, KVANKT; Sp25, VANK; MDPLys, Mur-L-Ala-D-iGlnβ-$(CH_2)_4NH_2$.

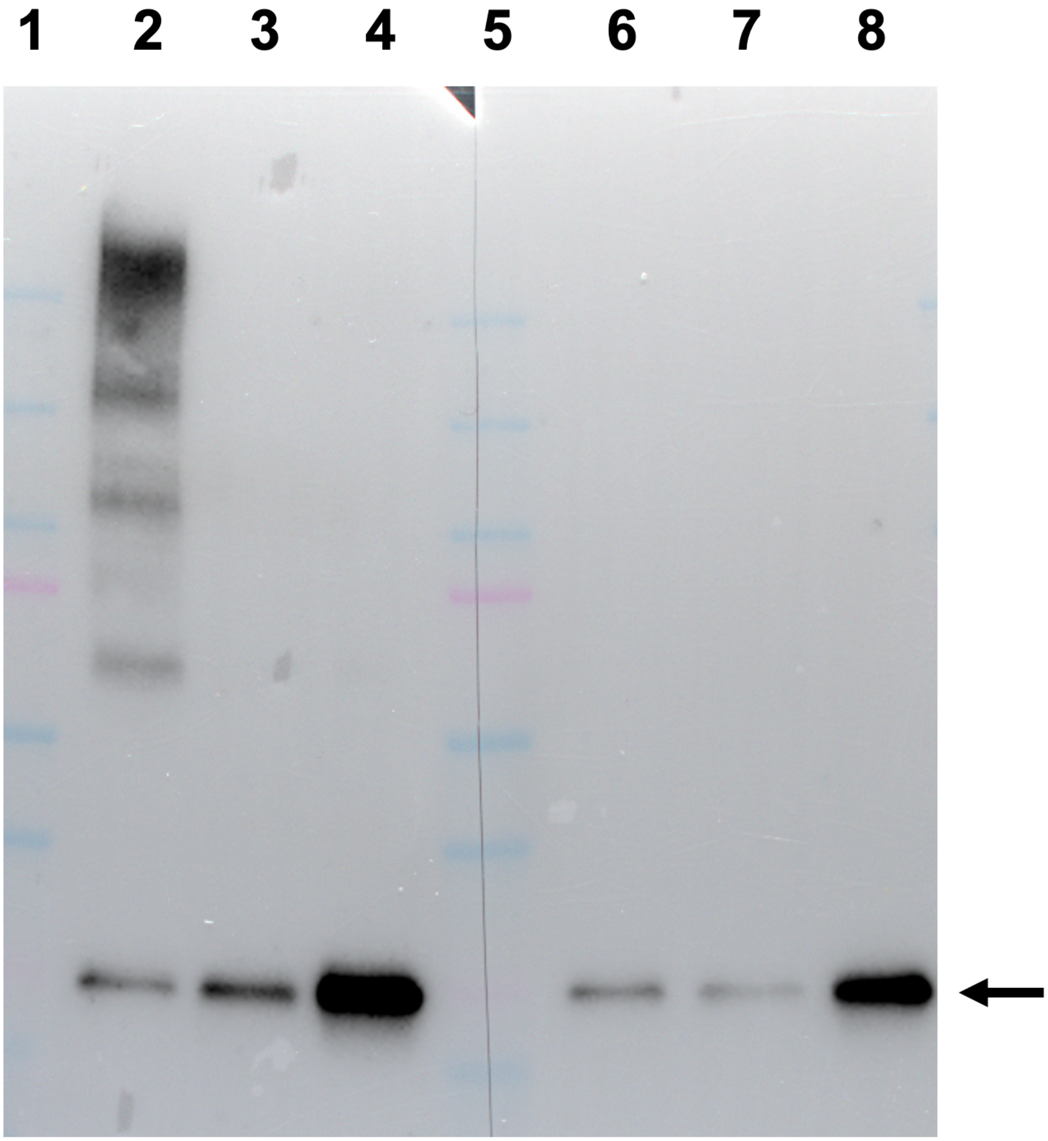

Supplementary Figure S1. Uncropped Western blot data for Figure 4a.

PLN lysates from various strains of mice (lanes 2 and 6, lysates from WT mice; lanes 3 and 7, lysates from GlcNAc6ST-1/-2 DKO mice; lanes 4 and 8, lysates from FucT-IV/-VII DKO mice) were blotted onto a PVDF membrane. The membrane was cut into half at lane 5 and probed separately with SF1 (lanes 2 to 4) or control IgG (lanes 6 to 8). Lanes 1 and 5, visible standard molecular weight marker. The blot was superimposed with the visible image to visualize the membrane cut and edges. Arrow indicates non-specific bands observed in the blot probed with control IgG.
